# Loss of Ten1 in mice induces telomere shortening and models human dyskeratosis congenita

**DOI:** 10.1126/sciadv.adp8093

**Published:** 2025-04-11

**Authors:** Adrián Sanz-Moreno, Lore Becker, Kan Xie, Patricia da Silva-Buttkus, Nathalia R. V. Dragano, Antonio Aguilar-Pimentel, Oana V. Amarie, Julia Calzada-Wack, Markus Kraiger, Stefanie Leuchtenberger, Claudia Seisenberger, Susan Marschall, Birgit Rathkolb, Enzo Scifo, Ting Liu, Anoja Thanabalasingam, Raul Sanchez-Vazquez, Paula Martinez, Maria A. Blasco, Sharon A. Savage, Helmut Fuchs, Dan Ehninger, Valérie Gailus-Durner, Martin Hrabê de Angelis

**Affiliations:** ^1^ Institute of Experimental Genetics, German Mouse Clinic, Helmholtz Zentrum München, German Research Center for Environmental Health (GmbH), Ingolstädter Landstraße 1, 85764 Neuherberg, Germany.; ^2^ Translational Biogerontology Lab, German Center for Neurodegenerative Diseases (DZNE), Venusberg-Campus 1/99, 53127 Bonn, Germany.; ^3^ German Center for Diabetes Research (DZD), Ingolstädter Landstraße 1, 85764 Neuherberg, Germany.; ^4^ Institute of Molecular Animal Breeding and Biotechnology, Gene Center, Ludwig-Maximilians-University München, Feodor-Lynen Str. 25, 81377 Munich, Germany.; ^5^ Telomeres and Telomerase Group–Fundación Humanismo y Ciencia, Molecular Oncology Program, Spanish National Cancer Centre (CNIO), Melchor Fernández Almagro 3, Madrid, E-28029, Spain.; ^6^ Clinical Genetics Branch, Division of Cancer Epidemiology and Genetics, National Cancer Institute, Bethesda, MD, USA.; ^7^ Chair of Experimental Genetics, TUM School of Life Sciences, Technische Universität München, Alte Akademie 8, 85354 Freising, Germany.

## Abstract

Telomere length regulation is essential for genome stability as short telomeres can trigger cellular senescence and apoptosis constituting an integral aspect of biological aging. Telomere biology disorders (TBDs) such as dyskeratosis congenita (DC) are rare, inherited diseases with known mutations in at least 16 different genes encoding components of the telomere maintenance complexes. The precise role of TEN1, part of the CST complex (CTC1, STN1, and TEN1), and the consequences of its loss of function in vivo are not yet known. We investigated the first viable murine model of *Ten1* deficiency created by CRISPR-Cas9–mediated exon 3 deletion. *Ten1* homozygous knockout mice present with telomere attrition, short life span, skin hyperpigmentation, aplastic anemia, and cerebellar hypoplasia. Molecular analyses revealed a reduction of proliferating cells, increased apoptosis, and stem cell depletion with activation of the p53/p21 signaling pathway. Our data demonstrate that Ten1 deficiency causes telomere shortening and associates with accelerated aging.

## Teaser

TEN1 telomerase capping complex subunit deficiency leads to accelerated aging, less proliferating cells, and reduced life span.

## INTRODUCTION

Aging involves a decline of physiological functions and accumulation of damage to cells and organs, thus giving rise to numerous age-related characteristics and pathologies (*1*). Accelerated aging is manifested in progeroid syndromes, which are a heterogeneous group of rare human conditions mimicking several aspects of natural aging such as hair loss, skin and bone alterations, reduced repair capacities, as well as impaired sensory and motor functions (*2*, *3*). Mechanisms of aging include cellular senescence, stem cell exhaustion, chromosome instability, and telomere attrition.

Eukaryotic telomeres are highly conserved DNA-protein complexes at the ends of linear chromosomes, protecting chromosomes during replication and preventing unwanted DNA repair mechanisms. To date, germline pathogenic variants (i.e., mutations) following autosomal dominant, autosomal recessive, or X-linked recessive inheritance, as well as de novo occurrence, have been reported in at least 16 different genes encoding components of telomere maintenance complexes as causes of the spectrum of telomere biology disorders (TBDs) (*4*).

The inability of DNA polymerase to fully replicate DNA ends results in telomere shortening with each cell division and vulnerability of chromosome ends. Cellular senescence or apoptosis is triggered when telomeres reach a critically short length to prevent continued cell division in an environment of accumulating DNA damage. Hence, telomere length acts as an inherent clock for cellular aging and proliferation capacity, being essential for genome stability and replication (*5*, *6*). Short telomeres are associated with a DNA damage response, resulting in the activation of different pathways, including p53 and p21 (*7*–*9*).

Dyskeratosis congenita (DC) and related TBDs are rare, tumor-prone, inherited bone marrow failure (BMF) syndromes caused by very short telomeres for age. DC is classically associated with the mucocutaneous triad of oral leukoplakia, nail dystrophy, and abnormal skin pigmentation. Individuals with DC/TBDs are at very high risk of BMF, pulmonary fibrosis, liver disease, and cancer (*4*, *10*, *11*). The clinical spectrum of TBDs is broad with childhood-onset disorders including DC features and other abnormalities such as cerebellar hypoplasia and immunodeficiency in Hoyeraal Hreidarsson syndrome and bilateral exudative retinopathy and intracranial calcifications in Revesz and Coats plus syndromes (*4*, *10*, *11*). Three main protein complexes are involved in telomere maintenance: telomerase, shelterin, and the CTC1-STN1-TEN1 (CST) complex. Telomerase is a ribonucleoprotein enzyme complex consisting of the telomerase reverse transcriptase (encoded by *TERT*), human telomerase RNA (hTR; encoded by *TERC*), and several accessory proteins. Shelterin, a six-protein complex that binds telomeric DNA, protects telomeres from the DNA damage response and modulates telomerase. Germline mutations in several shelterin components are associated with TBDs (*4*, *12*).

The CST complex, composed of three proteins in mammals (CTC1, STN1, and TEN1), is highly conserved in structure and function (*13*). It binds to single-stranded DNA (ssDNA), aids in C-strand synthesis, and limits telomerase stimulation by sequestration of the ssDNA substrate or binding to POT1-TPP1 of the shelterin complex (*14*). While CTC1 is the main binding site for G-overhangs, it also binds to STN1, a protein that is suggested to assist DNA polymerases in C-strand fill-in (*15*). TEN1 binds to STN1 as well and is indispensable for stabilizing the complex and thus for telomere maintenance, but it is also proposed to play a crucial role in C-strand fill-in (*16*, *17*). There is evidence that the CST complex forms oligomeric supercomplexes (*18*), suggesting that its precise conformation is important for CST function. In human cells, TEN1 plays an important role in genome-wide replication restart (*19*), by protecting stalled replication forks from degradation and thus maintaining chromosomal stability beyond telomeres (*20*).

Biallelic germline mutations in CTC1 and rarely in STN1 cause Coats plus syndrome, a pleiotropic multisystem disorder (*21*, *22*). CTC1 mutations have also been linked with classic DC (*23*, *24*) and shown to compromise telomeric DNA replication (*25*). Coats plus patients with mutations in STN1 and a zebrafish model that recapitulates the disease were described (*22*). To date, no patients with *TEN1* mutations have been reported.

In this study, we created and analyzed the first viable murine model of TEN1 deficiency associated with failure to thrive, reduced life span, and several hallmarks of DC, Hoyeraal-Hreidarsson syndrome, and related TBDs. We show that TEN1 ablation leads to shortened telomeres as well as decreased cell proliferation, apoptosis induction, activation of p53/p21, and increased inflammation in several organs. This model advances the understanding of TEN1 function by allowing detailed investigations of the organ-specific and time-dependent pathologies.

## RESULTS

### Severe failure to thrive and reduced life span observed in *Ten1* homozygous mice

**Fig. 1.**
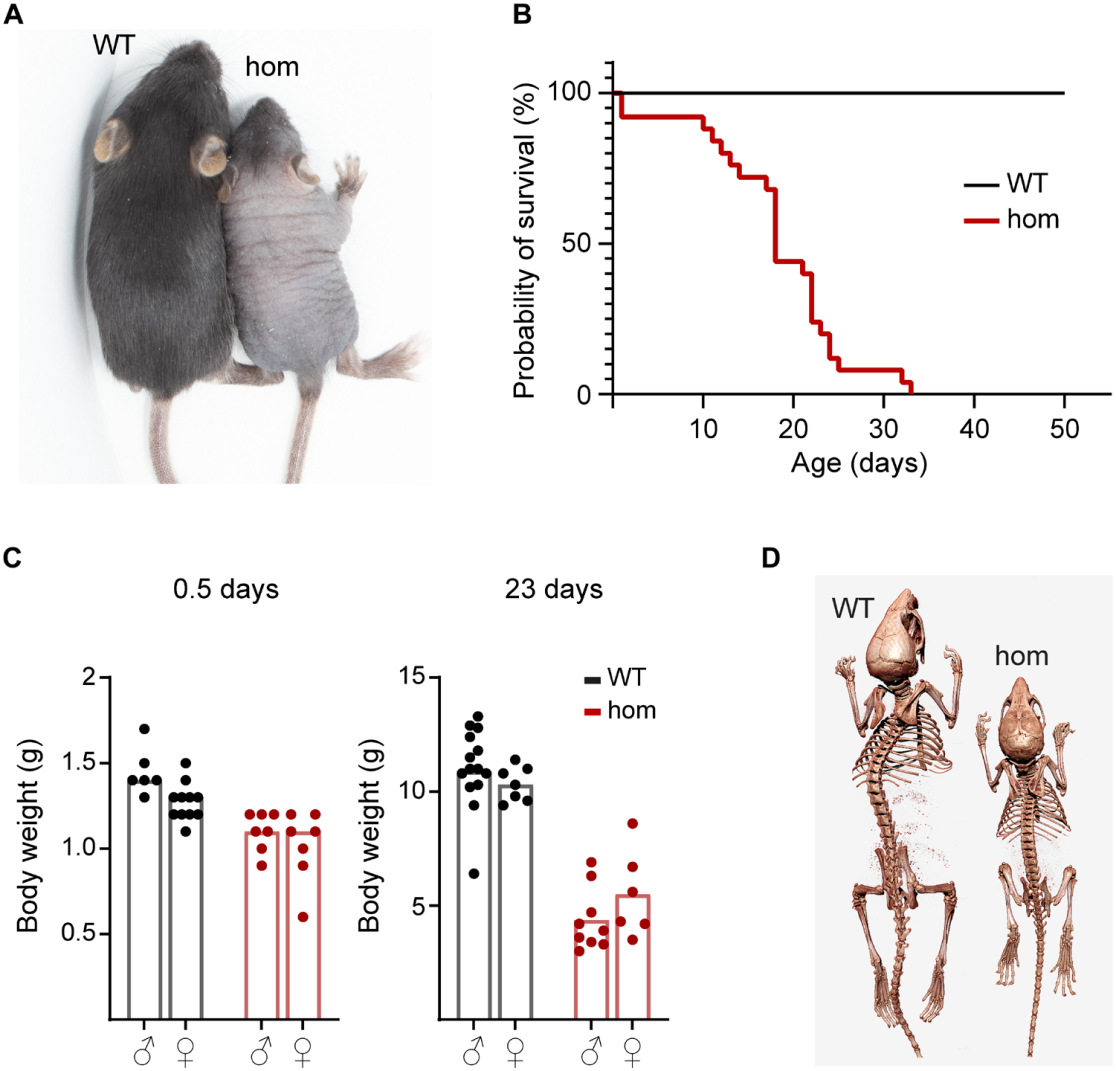
Appearance, body weight, survival rate, and telomere length of *Ten1* hom mice. (**A**) Body size and appearance in *Ten1* hom mice at P23. (**B**) Survival analysis (Kaplan-Meier: *P* < 0.0001; *n* WT/hom: 25/29). (**C**) Body weight at P0.5 and P23 (unpaired *t* test: *P* < 0.0001 each; *n* P0.5 WT/hom: 17/18, P23 WT/hom: 21/15). (**D**) μCT imaging for skeleton analysis.

We generated a *Ten1* knockout mouse mutant using CRISPR-Cas9 technology in the context of the International Mouse Phenotyping Consortium (IMPC; www.mousephenotype.org) resulting in the deletion of major parts of exon 3 (fig. S1A). Sequencing of the RNA quality control (QC) product predicted the first 30 amino acids of TEN1 to be translated followed by a frameshift and a premature stop after 87 amino acids. The absence of wild-type (WT) *Ten1* mRNA transcript in homozygous (hom) mice was confirmed by real-time quantitative polymerase chain reaction (RT-qPCR; fig. S1B). Mice were born close to an expected Mendelian genotype distribution, irrespective of sex (*P* = 0.473; chi-square test; fig. S1C). At birth, postnatal day 0.5 (P0.5), no obvious abnormalities were detected, although *Ten1* hom mice exhibited slightly reduced size. However, hom mice soon developed severe failure to thrive, alopecia, and a shortened life span (Fig. 1, A and B). These phenotypes were fully penetrant; therefore, data from both sexes were combined for further analyses. Heterozygous (het) *Ten1* mice did not show any of the phenotypes observed in hom animals up to 1 month of age. The mean survival of hom mice was 18.3 ± 7.7 days, with some animals dying early and only a few reaching an age of 32 days, compared to a minimum observation time of 50 days for their WT littermates (*P* < 0.0001; *n* WT/hom = 25/29; Fig. 1B). Mean body weight was already reduced at P0.5 in hom mice, but differences further increased at P20 to P23 (Fig. 1C). No skeletal abnormalities were detected using micro-computed tomography (μCT) at P26 (Fig. 1D), except for the size differences.

**Fig. 2.**
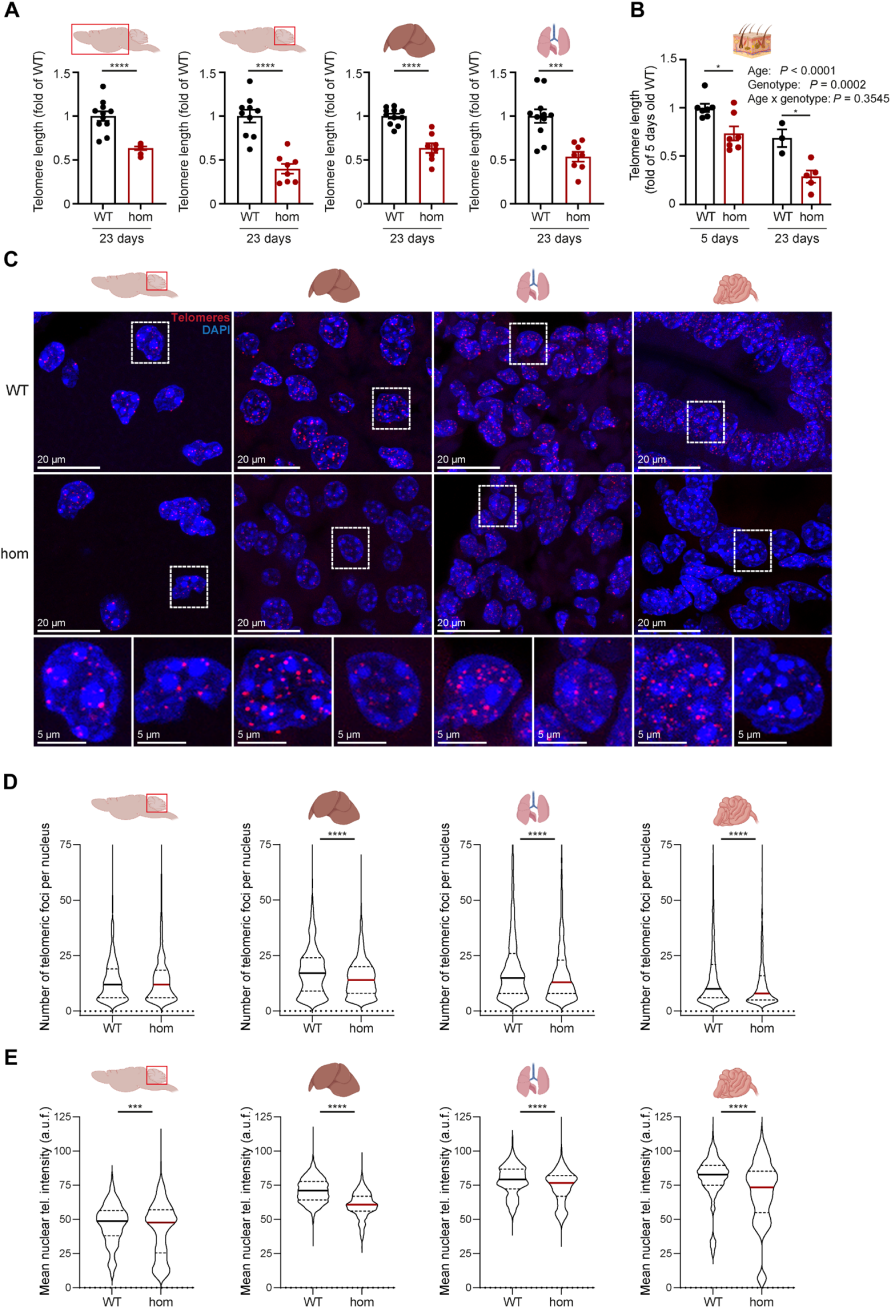
Telomere length analysis. (**A**) Telomere length by qPCR in the cerebrum, cerebellum, liver, and lung of *Ten1* hom animals at 23 days of age. (**B**) Telomere length by qPCR at P5 and P23 in the skin of *Ten1* WT and hom animals. (**C**) Representative images of Q-FISH immunofluorescence staining in the cerebellum, liver, lung, and small intestine at P23. The insets show magnification images of WT (left) and hom (right) cells below each tissue. Violin plots showing the (**D**) number of telomeric foci per nucleus and (**E**) mean nuclear telomeric intensity determined by Q-FISH in the cerebellum, liver, lung, and small intestine of *Ten1* WT and hom P23 animals (*n* WT/hom: 5/5). a.u.f., arbitrary units of fluorescence. Three to four images corresponding to different areas of the tissues were analyzed from each mouse. The median and the upper and lower quartiles of each distribution are indicated. Statistical significance was addressed by two-tailed Student’s *t* test. *P ≤ 0.05; ***P ≤ 0.001; ****P ≤ 0.0001. Figure elements were created with BioRender.

Telomere length analyzed by qPCR was reduced in homs compared to controls across various tissues, including the cerebrum, cerebellum, liver, and lung (Fig. 2A). Analysis of telomere length by qPCR in skin at different ages also revealed an age-related decline (Fig. 2B). We observed a significant reduction in the relative abundance of telomeric sequences in all the *Ten1*-deficient tissues investigated, as compared to WT controls, indicative of decreased average telomere length in hom mice.

To analyze the potential differences in telomere length distributions between WT and *Ten1* hom mice, we performed telomeric quantitative telomere fluorescence in situ hybridization (Q-FISH) analysis in the cerebellum, liver, lung, and small intestine tissues at P23 (Fig. 2, C to E). The results showed a significant lower number of detected telomere foci per nucleus in the liver, lung, and intestine but not in the cerebellum, indicative of the possible presence of very short undetectable telomeres in *Ten1* hom mice (Fig. 2D). The distribution of the mean nuclear telomere intensity in the four tissues analyzed clearly showed significantly decreased median and lower quartile values in knockout cells of the four tissues analyzed (Fig. 2E). The mean nuclear telomere length decreased by 8, 15, 6, and 23% in the cerebellum, liver, lung, and small intestine of *Ten1* hom mice compared to controls, respectively. These results demonstrate reduced telomere length as well as telomere loss in hom mice, underscoring a role of TEN1 in telomere length maintenance. The levels of CTC1 and STN1 remained unchanged in all *Ten1* hom tissues examined by mass spectrometry when compared to controls (fig. S2).

### Skin hyperpigmentation, hair loss, tongue hyperkeratosis, and intestinal atrophy in *Ten1* hom mice

**Fig. 3.**
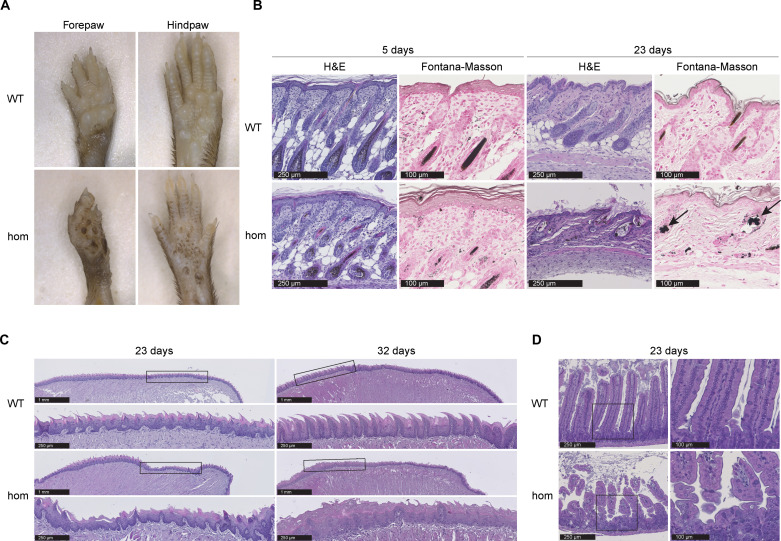
Skin hyperpigmentation, tongue hyperkeratosis, and intestinal atrophy in *Ten1* hom animals. (**A**) Hyperpigmentation in palmar and plantar aspects of the fore- and hindpaws of *Ten1* hom mice at P23 (*n* WT/hom: 5/9). (**B**) H&E staining of *Ten1* hom snout (P5) and flank (P23) skin. Melanin deposition demonstrated by Fontana-Masson staining (see arrows). Five days (*n* WT/hom: 2/4) and 23 days (*n* WT/hom: 6/6). (**C**) Tongue H&E staining at P23 (*n* WT/hom: 6/7) and P32 (*n* WT/hom: 1/1) showed hyperkeratosis, decreased number of papillae, architectural disorganization, and nuclear pleomorphism in *Ten1* hom mice. (**D**) Decreased villi length and signs of mucosal atrophy in the small intestine of *Ten1* hom mice at P23 (*n* WT/hom: 6/6).

The hom mutants exhibited reduced hair growth as well as smaller size and became easily distinguishable from their littermate controls starting around P10 (fig. S3). Hyperpigmentation in the palmar and plantar surfaces of the fore- and hindpaws was found in *Ten1* hom mice of both sexes at P23 with walking pads being particularly affected (Fig. 3A). Examination of hematoxylin and eosin (H&E)–stained snout and flank skin sections at P5 and P23 evidenced a progressive accumulation of pigment in *Ten1* hom mice, mainly in hair follicle remnants (Fig. 3B) but also focally in the epidermis. Fontana-Masson silver stain confirmed that this pigment was melanin (arrows in Fig. 3B). Collectively, *Ten1* hom flank skin dermis and hypodermis appeared thinner than in WT animals at P23 and contained obvious hair follicle remnants (in agreement with the macroscopic observation of alopecia) filled with melanin (Fig. 3B).

Since many of the phenotypes mentioned above resemble disease features associated with DC, *Ten1* hom mice were examined for oral leukoplakia, another pathological characteristic in patients with DC. No visible white plaques were observed macroscopically on the surface of the tongue in mutants. However, H&E staining revealed hyperkeratosis, fewer papillae, architectural disorganization, and nuclear pleomorphism in *Ten1* hom mice at P23 and P32 (Fig. 3C). We also evaluated the small intestine of *Ten1* hom mice and detected decreased villi length and signs of mucosal atrophy at P23 (Fig. 3D).

### BMF and thymic atrophy in *Ten1* hom mice

**Fig. 4.**
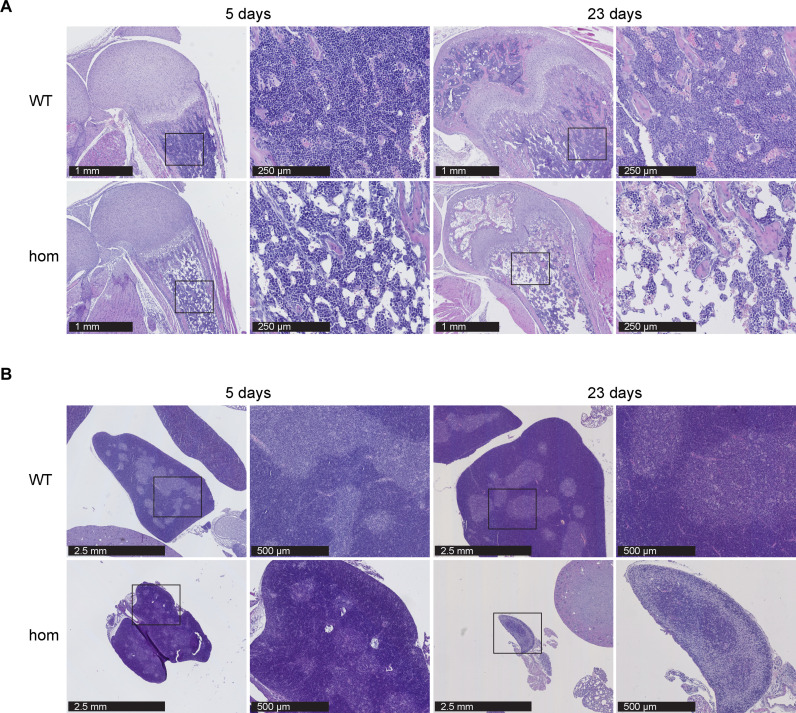
BMF (aplastic anemia) and thymic atrophy in *Ten1* hom mice. (**A**) H&E-stained femoral bone marrow at P5 and P23 of control and hom mice (P5 *n* WT/hom: 2/3; P23 *n* WT/hom: 3/3). (**B**) Thymic atrophy in P23 *Ten1* hom animals (P5 *n* WT/hom: 2/7; P23 *n* WT/hom: 6/7).

H&E staining of femoral bone marrow in *Ten1* hom mice showed alterations typical of aplastic anemia, characterized by the replacement of hematopoietic cells (including erythroid, lymphoid, and myeloid progenitors) with stromal adipose tissue at P23 (Fig. 4A). Microscopic analysis of the bone marrow at P5 showed only a slight reduction in the number of hematopoietic cells in knockout mice, pointing to a progressive hematological defect (Fig. 4A). As shown in Fig. 1, growth retardation was obvious in *Ten1* hom mice, and we observed notably less hypertrophic chondrocytes, crucial for endochondral ossification, in the tibial growth plates of *Ten1* hom mice from P5 to P23 (fig. S4). In addition, thymic atrophy was observed as another major immune system defect in *Ten1* hom mice at P23, with decreased cellularity of the cortex (appearing pale) and a dark basophilic medulla (Fig. 4B). These atrophic changes in the thymus were not obvious at P5 in *Ten1* hom mice (Fig. 4B).

### Neurologic abnormalities, cerebellar hypoplasia, and retinal degeneration after *Ten1* deletion

**Fig. 5.**
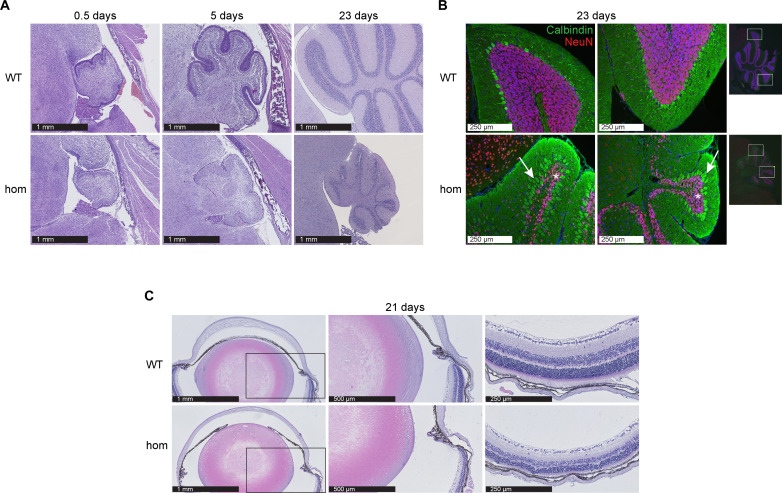
Cerebellar hypoplasia and retinal degeneration in *Ten1* hom mice. (**A**) Representative pictures of H&E-stained cerebellum sections at P0.5 (*n* WT/hom: 6/6), P5 (*n* WT/hom: 5/8), and P23 (*n* WT/hom: 6/5) at the same magnification. (**B**) Double immunofluorescence of granular (asterisk; NeuN, red) and Purkinje (arrow; calbindin, green) cells in the cerebellum of *Ten1* hom animals at P23 (*n* WT/hom: 4/4). (**C**) Ocular changes in P21 *Ten1* hom animals: Anterior synechia and retinal alterations at P21 are displayed (*n* WT/hom: 3/5).

After birth, the behavior of pups first appeared normal, but reduced locomotion was observed starting around P10, with *Ten1* hom mice beginning to show tremors, ataxic movements, and occasional seizures. Brain histology showed a pronounced reduction in cerebellar size (cerebellar hypoplasia) in *Ten1* hom mice, particularly evident at P23 (Fig. 5A). Double immunofluorescence showed decreased granular neuron (NeuN-positive) cell number (Fig. 5B). Instead of one monolayer of well-ordered Purkinje cells (calbindin-positive) as observed in controls, several layers of cells were often found stacked together in a disorganized fashion in *Ten1* hom mice at P23 (see arrows in Fig. 5B).

Notable ocular phenotypes were also observed. These included a thinner cornea, anterior synechia (adhesion between the iris and cornea), and reduced total retinal thickness in *Ten1* hom mice at P21 (Fig. 5C). At P9, the iris was completely attached to the cornea in hom mice, resulting in severe synechia. However, the iris and the cornea appeared to be almost separate by P21, indicating an improvement in the severity of synechia in *Ten1* hom mice (Fig. 5C, left and middle). In contrast, the maldeveloped retinal layers phenotype showed little change over time, with persistent reduction in retinal thickness and fewer cell nuclei in both the outer and inner nuclear layers (Fig. 5C, right). The retina also exhibited rosette-like alterations, which seemed to progress in severity from P9 to P21 in *Ten1* hom mice. Table 1 shows a summary of the phenotypes present in *Ten1* hom mice together with a comparison of symptoms seen in human DC.

**Table 1.** Comparison of human TBD clinical manifestations and *Ten1* knockout mouse phenotypes. Reported frequencies in NCI DC/TBD cohort modified from (*10*). Symptoms with less than 5% frequency were omitted. Hoyeraal-Hreidarsson syndrome modified from (*50*). KO, knockout; n.d., not detected; NA, not analyzed; BMD, bone mineral density.

Human manifestations	Reported frequency in NCI DC/TBD cohort (%)	Hoyeraal-Hreidarsson syndrome	*Ten1* KO mouse phenotype	Additional mouse phenotypes
Classical triad (min. 2 of 3)	42.9			
Skin reticular pigmentation		++	Yes	
Nail dystrophy		+	n.d.	
Oral leukoplakia		++	Yes	
			Yes	Alopecia
Hematopoiesis				
BMF	61.6	+++	Yes	
Immunodeficiency	35.7	++	Yes	Thymic atrophy
Growth				
Intrauterine growth retardation	32.1	+++	Yes	
Short stature	6.6		Yes	Bone growth
Neurological features				
Microcephaly	20.7	++	n.d.	
Cerebellar hypoplasia	37.3	+++	Yes	
Developmental delay	28.6	+++	Yes	
Ataxia	15.4	+	Yes	
Ophthalmologic abnormalities				
Epithelial	27.5		Yes	Anterior synechia, corneal thinning
Retinal	8.5	+	Yes	Retinal degeneration
Dental abnormalities	30		n.d.	
Pulmonary fibrosis	6.6		n.d.	
Gastrointestinal				
Failure to thrive	8.8	++	Yes	
Esophageal strictures	7.7	++	n.d.	
other		+	Yes	Intestinal atrophy
Genitourinary system (urethral stenosis)	20.8		n.d.	
Endocrine system	13.2		NA	
Skeletal system				
Osteoporosis	31.6		Subtle	2/4 male homs: lower BMD

### Reduced cell proliferation, increased apoptosis, and signs of stem cell depletion in *Ten1* hom mice

**Fig. 6.**
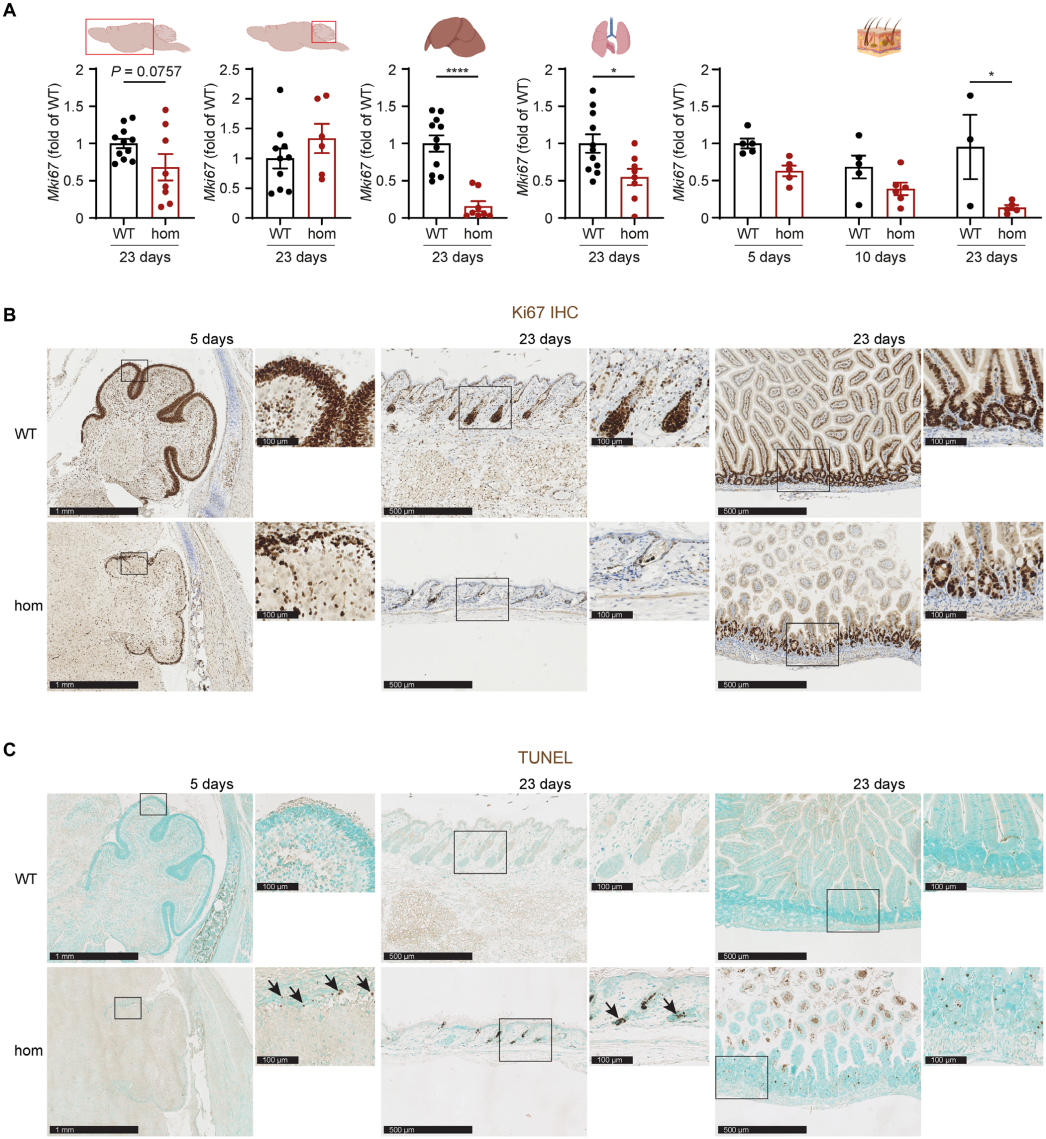
Analysis of proliferation and apoptosis in *Ten1* hom animals. (**A**) *Mki67* gene expression levels for cell proliferation analysis in the cerebrum, cerebellum, liver, lung, and skin at P23. *P ≤ 0.05; ****P ≤ 0.0001. (**B**) IHC showed fewer Ki67-positive cells in *Ten1* hom mice compared to controls in cerebellum at P5 and in skin and small intestine at P23. (**C**) TUNEL staining for apoptosis in *Ten1* hom animals in cerebellum at P5 and in skin and small intestine at P23. Figure elements in (A) were created with BioRender. For (B) and (C): *n* WT/hom: P5 2/4; P23 6/7.

Since telomere maintenance and chromosomal stability are crucial for cell survival and proliferation capacity, a set of markers for proliferation, cell cycle, and apoptosis was analyzed. *Mki67* encodes for the protein Ki67, necessary for maintenance of mitotic chromosomes and commonly used as proliferation marker. RT-qPCR analyses unveiled lower expression of *Mki67* in *Ten1* hom mice when compared to controls in the liver, lung, and skin at P23 (Fig. 6A). A similar trend was observed in the cerebrum although the difference did not reach statistical significance (*P* = 0.0757; Fig. 6A). *Mki67* expression in the cerebellum at P23 was not significantly different between genotypes (Fig. 6A). In addition, immunohistochemistry (IHC) revealed a lower number of Ki67-positive (proliferating) cells in cerebellum at P5 and skin and small intestine at P23 (Fig. 6B) in *Ten1* hom mice compared to WT. Expression of cell cycle progression regulating cyclin genes was also measured in various tissues (tables S1 and S2). The transcriptional patterns of cyclin genes in the liver, lung, and skin suggest reduced proliferative capabilities in *Ten1* hom mice at P23 (tables S1 and S2). On the other hand, Ten1 deletion triggered elevated expression of some cyclin genes (*Ccna1*, *Ccna2*, *Ccnd2*, and *Ccne1*) in the cerebellum, whereas others (*Ccnb2* and *Ccne2*) showed a significant decrease in the mutants (table S1).

Apoptosis, as determined by terminal deoxynucleotidyl transferase–mediated deoxyuridine triphosphate nick end labeling (TUNEL) staining, was increased in the *Ten1* hom cerebellum at P5 and in skin and crypts of the small intestine at P23 (Fig. 6C). To analyze possible stem cell depletion or exhaustion, IHC for the stem cell markers Sox9 and CK15 was performed (*26*–*28*) and revealed a progressive loss of Sox9-positive cells in *Ten1* hom skin and small intestine at P32, while differences in the cerebellum were already obvious at P5 (fig. S5A). Analysis of CK15 by IHC in flank skin also showed a similar tendency in *Ten1* hom mice at P32 (fig. S5B).

### Activation of p53 and p21 pathways in several organs of mutant mice

**Fig. 7.**
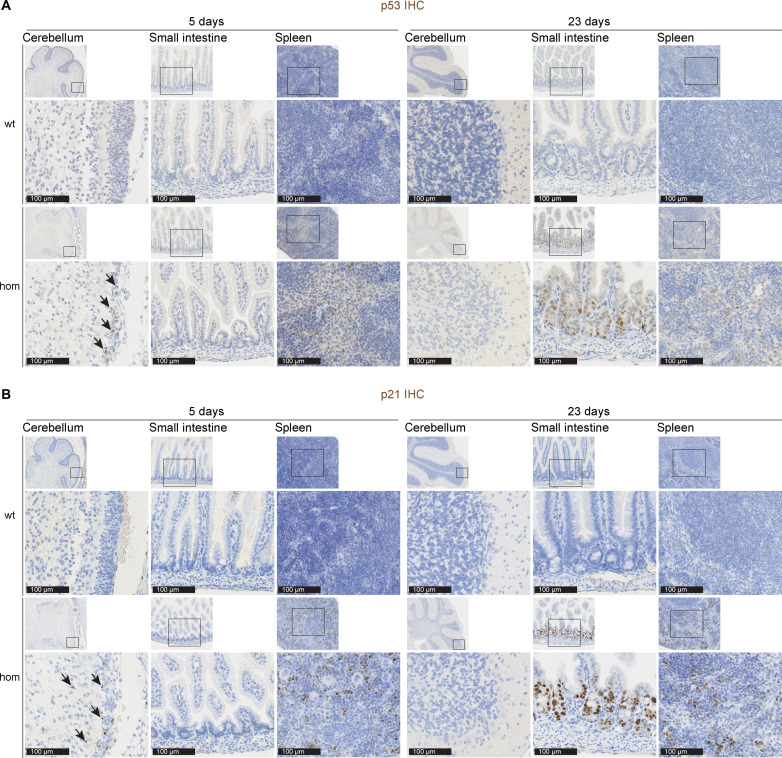
Analysis of p53 and p21 expression in *Ten1* hom mice. Representative pictures of p53 (**A**) and p21 (**B**) IHC in the cerebellum, small intestine, and spleen from control and *Ten1* hom mice at P5 (left) and P23 (right). The area inside the rectangles of the low magnification pictures on top is shown below on a higher magnification. For (A) and (B): *n* WT/hom: P5 2/4; P23 6/7.

IHC of p53 (Fig. 7A) and downstream cell cycle arrest protein p21 (Fig. 7B), for evaluation of possible cell stress responses, showed that both were highly expressed in *Ten1* hom mice at P5 in the cerebellum, at P23 in the small intestine and at these two time points in the spleen. For other organs, RT-qPCR showed increased *Trp53* (encoding p53) expression in cerebellum at P23 (fig. S6A). Also, *Cdkn1a* (encoding p21) was found up-regulated in *Ten1* hom cerebrum, liver, lung, and skin, when compared to WT (fig. S6B).

In addition, several cellular senescence-associated transcripts (*p16Ink4a*, *p19Arf*, *Bcl2*, *Bhlhe40*, and loss of *Lmnb1*) established in the current literature were assessed by RT-qPCR. From all tissues and markers analyzed in fig. S7, an induction of senescence was observed in skin (*p16Ink4a* and loss of *Lmnb1*), cerebrum (*p19Arf* and loss of *Lmnb1*), liver (*Bcl2*, *Bhlhe40*, and loss of *Lmnb1*), and lung (*Bcl2*) in *Ten1* hom mice versus WT at P23. In contrast, a significant inhibition of senescence in hom mice at P23 was only found in the liver (*p16Ink4a* and *p19Arf*) (fig. S7). Collectively, senescence markers were up-regulated in several tissues of *Ten1* hom mice at P23, although to a variable extent.

### Mild DNA damage response detected in P23 *Ten1* hom animals

TEN1 has an important role in genome-wide replication (not only in the telomere). As replication defects are often followed by a DNA damage response and we observed p53/p21 activation, we analyzed the expression of the DNA damage marker phospho-histone H2AX (Ser^139^) using Western blot (WB) and IHC (fig. S8, A to C). No obvious differences in pH2AX were detected in the liver, lung, and cerebrum by WB (fig. S8, A and B). By IHC, we analyzed a wider collection of tissues at P23 that mostly showed no pH2AX positive cells. We could only see some pH2AX positivity in the crypts of the small intestine of *Ten1* hom mice (fig. S8C). Also, very few cells were positive in the thymus and spleen of *Ten1* hom animals (fig. S8C). We also investigated key DNA repair proteins in a subset of the tissues and observed increased pATM (phospho-serine-protein kinase ATM) in small intestine by IHC (fig. S8D) and pATR (phospho-serine/threonine-protein kinase ATR) in liver by WB (fig. S8, E and F). No differences in pATM or pATR abundance were detected in lung (fig. S8, E and F). Together, we observed a mild DNA damage response (higher pH2AX and pATM in the small intestine and elevated pATR in the liver) in P23 *Ten1* hom animals.

### Increased expression of pro- and anti-inflammatory markers in multiple tissues of *Ten1* hom mice

Gene expression levels of a range of proinflammatory cytokines (*Ifng*, *Il1b*, *Il6*, and *Tnf*) as well as the chemokine *Ccl2* were analyzed in the cerebrum, cerebellum, liver, lung, and skin of *Ten1* hom versus WT mice at P23. In general, *Ten1* hom mice featured higher expression of proinflammatory markers. Expression of all four proinflammatory cytokines tested was up-regulated in the cerebrum (fig. S9, A to D). Similar patterns were observed in the cerebellum, liver, lung, and skin, although not all parameters examined reached statistical significance (fig. S9). However, *Tnf* expression in the lung was lower in *Ten1* hom compared to WT animals. *Ccl2* expression was significantly up-regulated in the cerebellum, liver, and skin in *Ten1* hom mutants, but no differences were found in the cerebrum and lung (fig. S9E). We also detected elevated gene expression levels of anti-inflammatory cytokines (*Il4*, *Il10*, and *Il13*) in many of these *Ten1* hom tissues at P23 (fig. S10).

### Elevated transcription of retrotransposons in the liver and lung of mutant mice

Whether DC is associated with an alteration of transposable elements has not been investigated yet, at least to our knowledge. We determined mRNA abundance of four retrotransposon classes [long interspersed nuclear element 1 (LINE1), MusD, B1, and B2] in *Ten1* hom versus WT mice via RT-qPCR. Transcription of all four classes of retrotransposons was elevated in *Ten1* hom liver and lung at P23, but no increase in transposon transcription was found in the cerebrum, cerebellum, and skin (fig. S11).

### None of the described phenotypes detectable in Ten1 het mice up to 1 month of age

In contrast to the various pathological findings *Ten1* hom mice, het mice up to 1 month of age did not show any of the described phenotypes (see representative pictures, body weight analysis, and selected organs of *Ten1* het mice in fig. S12).

### TEN1 conservation across species and apparently no tolerance of pathogenic variants in humans

Human *TEN1* is a relatively small gene with four exons across 21,347 nucleotides, encoding 123 amino acids and six transcripts (GRCh37.p.13, chromosome 17: 73,975,321 to 73,996,667). Alignments of human TEN1 protein with six other species showed 100% homology with chimpanzee, 81.8% with dog, 67.2% with rat, 62.3% with mouse, 51.2% with *Xenopus*, and 42.5% with zebrafish (fig. S13, A and B).

There were no germline variants of interest in patients with DC in the National Cancer Institute Inherited Bone Marrow Failure Syndromes (NCI IBMFS) or the UK DC cohorts. In addition, a query of the Undiagnosed Disease Network cases did not identify TEN1 variants of relevance. There were no pathogenic, likely pathogenic, or variants of uncertain significance reported in ClinVar’s 30 April 2023 release. Two hundred five TEN1 variants were present in 141,456 Genome Aggregation Database (gnomAD) individuals across all ethnic groups. Only two variants had a minor allele frequency (MAF) >0.01 (c.198C > T, p.His66His and c.136G > T, p.Ala46Ser). There were 177 variants with MAF < 0.0001. Constraint metrics calculated by gnomAD suggest that TEN1 is tolerant of synonymous single-nucleotide variants based on observed/expected (o/e) = 1.03 [90% confidence interval (CI) 0.77 to 1.39]. However, it appears to be intolerant of missense variation (o/e = 0.75, 90% CI 0.6 to 0.94) and loss of function variation (o/e = 0.67, 90% CI 0.3 to 1.61).

## DISCUSSION

Telomere maintenance and chromosomal stability are crucial for development, growth, and survival of mammalian cells and organisms. Disrupting any of the players of the well-orchestrated and complex machineries involved has a high potential for severe vital consequences. TEN1 is the smallest subunit of the heterotrimeric CST complex, and, to date, no organism with a loss-of-function mutation has been described in the literature. While mouse telomeres are considerably longer than those of humans, their telomere biology is similar and common mechanisms and manifestations can be analyzed in mice (*29*, *30*). Here, we show that ablation of *Ten1* in mice is sufficient to cause telomere shortening, growth retardation, BMF, early lethality, and many features of human TBDs. Neither CTC1 nor STN1 was up-regulated in our model, suggesting that loss of *Ten1* was not compensated for in this model, which is in line with findings obtained in a previous study (*17*).

The phenotypes observed in our *Ten1* loss-of-function model are markedly similar to the spectrum of clinical manifestations seen in DC in general and HH syndrome in particular. Our findings are in agreement with a progressive telomere-mediated disease that primarily affects high turnover tissues (*31*). Alopecia has been described in other mouse models of TBDs (*32*, *33*), and studies in humans suggest a causal relationship between telomere length and androgenetic alopecia (*34*, *35*). Skin hyperpigmentation is common in aging skin as well as in TBDs in mice (*36*, *37*) and humans (*38*, *39*) and can be induced by p53 stimulation (*40*). Thinner skin, aberrant pigmentation, and accumulation of senescent cells (expressing p16) are hallmarks of aging human skin, and these features are also present in *Ten1* hom animals (*41*, *42*).

The nail dysplasia of patients with DC (*43*) was not detected in *Ten1* hom mice, probably due to the short life span of our model. Likewise, tongue hyperkeratosis was observed in our mice, but severity did not reach oral leukoplakia as seen in human patients with DC (*44*). Analysis of oral leukoplakia as precancerous lesions in humans revealed reduced telomere length in affected and surrounding cells (*45*). Gastrointestinal pathology, with increased intestinal cell death, has been described in telomere-mediated disease in general and DC in particular (*46*). Compatible with our findings, intestinal atrophy was also reported in mice with shorter telomeres or telomerase deficiency (*47*–*49*).

The progressive BMF observed might be crucial for the early death of *Ten1* hom animals, comparable to HH patients (*50*). Together with the thymic atrophy, also present in other telomere dysfunction mouse models, these observations are consistent with progressive hematological and immunological phenotypes in *Ten1* hom animals. Chondrocyte hypertrophy is a key step in endochondral ossification leading to proper longitudinal bone growth (*51*). Thus, the decrease in the number of hypertrophic chondrocytes in *Ten1* hom growth plates over time could be associated with their reduced body size during postnatal development.

The earliest pathological manifestation detected in *Ten1* hom mice was cerebellar hypoplasia, a hallmark of HH syndrome (*50*), already seen at P5 in our mice, in agreement with the ataxia phenotype observed. TBDs, predominantly Revesz syndrome and Coats plus, have also been associated with several eye-related conditions, such as exudative retinopathy, and peripheral pinpoint retinal pigment epithelial detachments (*52*, *53*). Within the CST complex, mutations in *CTC1* and *STN1* have been established as contributors to ocular manifestations, such as exudative retinopathy and retinal telangiectasia, which have been frequently observed (*21*, *54*). The *Ten1* mouse model presented here broadens the spectrum of ocular findings attributed to the CST complex. These additional effects encompass a thinner cornea, anterior synechiae (iris-corneal adhesion), and retinal degeneration.

Aging is associated with reduced proliferative capabilities (*55*, *56*) and dysregulation of cellular apoptotic activities (*57*). In addition, stem cell depletion is a hallmark of aging (*58*, *59*) and has been associated with DC (*60*, *61*). Our data are consistent with the “stem cell defect” that has been proposed in the context of telomere dysfunction and associated with phenotypes observed in highly proliferative tissues (as bone marrow, intestine, or skin) (*61*, *62*) as well as in mouse models of other defective genes important for telomere maintenance (*48*, *62*).

*Ten1* ablation is sufficient to cause telomere attrition and p53 activation. Dependent on the degree of hyperactivation, p53 has an important role in developmental syndromes by decreasing proliferation and inducing apoptosis, affecting survival and causing neuronal (including cerebellar hypoplasia), hematopoietic, pigmentation, or premature aging defects (*63*). Hyperactive p53 has been linked to features suggestive of DC in mice (*64*, *65*) and the p53/p21 pathway reported active in DC cells with telomerase insufficiency (*66*). A mutation in MDM4, a negative regulator of p53, was detected in patients with TBD, and generation of a mouse model of this mutation (p.T454M) recapitulated the DC phenotypes (*65*), in accordance with mice lacking the p53 C terminus showing TBD phenotypes (*64*).

Telomere shortening is a crucial inducer of cellular senescence, a state in which the cell undergoes cell cycle arrest but becomes more resilient against apoptosis and oncogenesis (*67*, *68*). We observed generally increased expression of senescence markers at P23 in *Ten1* hom mice, but the differences were subtle in the tissues and time points analyzed. The explanation for this is now unclear. A time-dependent switch from apoptosis to senescence was shown in telomerase-deficient zebrafish (*69*). It has also been suggested that low levels of p53 promote cell cycle arrest and senescence, while hyperactivation of p53 would lead to apoptosis (*70*).

Chronic inflammation as a systemic manifestation was coined as a hallmark of aging (*71*). While inflammatory activation in TBDs has only been documented in a few clinical cases (*72*, *73*), the general association between telomere dysfunction and chronic inflammation is well established in a variety of human pathological disorders, including cardiovascular, gastrointestinal, hepatic, infectious, neurological, and pulmonary diseases (*74*, *75*). Several clinical and preclinical studies support the notion that telomere dysfunction may drive inflammatory activation (*76*–*78*). On the other hand, it has been shown that chronic inflammation may induce telomere dysfunction and exacerbate a range of aging phenotypes in mice (*79*). Both lines of evidence combined point toward the potential existence of a feedback loop between telomere dysfunction and chronic inflammation that is mechanistically mediated by a number of cytokines [e.g., tumor necrosis factor–α, interleukin-1β (IL-1β), IL-6, IL-10, and interferon-γ] and chemokines (e.g., MCP-1) (*80*). In general, *Ten1* hom mutant mice feature inflammatory activation across tissue types which is in line with findings obtained from other mouse models of telomere dysfunction (e.g., *Ercc1* or *Terc*-deficient animals) (*74*, *76*).

Only about 1.5% of the entire mammalian genome encodes for proteins (*81*, *82*). A substantially higher proportion of the human (~45%) and the mouse (~37%) genomes are composed of repetitive DNA sequences, including LINEs, short interspersed nuclear elements, and long terminal repeats (*83*–*85*). Cellular mechanisms suppressing transposition events may be compromised in disease states [e.g., cancer (*86*)]. Transposable elements become more active at old age or after infection in mice (*87*, *88*). One physiological consequence demonstrated by multiple studies is the induction of systemic inflammation after retrotransposon activation (*89*, *90*). In *Ten1* hom mutant mice, we detected elevated transcriptional activities of transposable elements in the liver and lung but not in the brain and skin, suggesting that the degree of genomic instability caused by *Ten1* removal may vary between tissue types in the mouse. Given that the profiles of inflammation and transposon transcription only align in a subset of tissues, it may indicate that enhanced transposon activity does not represent the sole molecular mechanism underlying systemic inflammation in *Ten1*-deficient mice.

Similar to the mouse model described here, *Ctc1* deletion in mice leads to acute telomere loss and subsequent cellular proliferation defects causing complete BMF and premature death at around 24 days of age (*91*). In contrast, telomerase knockout mice have been shown to exert a phenotype only after several generations (*92*, *93*). The mechanistic basis for the marked difference in telomere shortening pace between *Ten1* or *Ctc1* and telomerase mouse knockouts is now not clear but might be derived from failure to maintain the telomeric C-strand and/or CST complex nontelomeric effects (crucial for maintaining genome stability) (*17*, *94*).

However, to what proportion telomeric defects underlie the pathologies we observed needs to be determined by future studies. Telomere length is reduced but probably not to an extent incompatible with life. Consistent with Feng *et al.* (*17*), we hypothesize that *Ten1* ablation leads to CST instability and thus reduced function. Since CST has been shown to also play a major role in overcoming replication fork stalling (*20*), the increased DNA damage and chromosomal instability observed in some tissues might also contribute to the phenotypes described.

The present work has some limitations. First, we have been unable to detect TEN1 at the protein level (either by using the only commercially available TEN1 antibody or by mass spectrometry). Thus, we cannot exclude the presence of a truncated and mutated version of TEN1 in hom mice (that might sequester other members of the CST complex). However, since no alterations were detected in het mutants so far, we suggest that there is no dominant negative effect of this *Ten1* mutation. Second, potential DNA replication defects should be further investigated to establish a clear mechanism of TEN1 function, as we only analyzed the role of TEN1 in telomeric maintenance and DNA damage in this model.

We propose that the *Ten1* mutation introduced impairs CST stability and function, thus reducing DNA maintenance processes. However, telomere shortening and DNA damage seem not so severe in the short life span of the mutant animals. We assume that BMF might be the main factor contributing to the early death observed. Future analyses of tissue-specific knockouts might allow for more mechanistic insights of TEN1 function in vivo.

To date, no human patients with TEN1 germline mutations have been described (*11*, *25*), suggesting a critical role of TEN1 for survival (*19*, *95*). Although TEN1 knockout cell lines have been reported (*17*), we found no evidence of viable eukaryotic organisms with *Ten1* deficiency in the literature. Still, the exact molecular mechanisms of TEN1 function(s) and the possible impact of human variants on aging-related phenotypes need to be further explored.

By investigating this mouse model, we shed light on the function of TEN1 in vivo and demonstrate the importance of the CST complex in telomere maintenance and viability. Our model allows a timely analysis of telomere shortening in vivo and may support the development of therapeutic interventions for TBDs and related pathologies.

## MATERIALS AND METHODS

### Mouse generation

The Ten1-em1/CRISPR-Cas [Ten1^em1(IMPC)Hmgu^] mouse model was generated using the IMPC targeting strategy with CRISPR-Cas technology (https://mousephenotype.org/data/genes/MGI:1916785) at Helmholtz Zentrum München in the context of the IMPC consortium, which aims to create a comprehensive catalog of mammalian gene function (*96*, *97*). For knockout generation, the web-based CRISPOR Design Tool (http://crispor.tefor.net/) was used, and four guides targeting 148 bp of *Ten1* exon3 and 179 bp 5′ intron sequence (ACCTACAACCTGCTTCACCCTGG; CCAGTGCATTCTCAATTTACAGG; GTTCTCACTACCATACGGATAGG; ACAGATCCGAACACAGCGTATGG; gene ID: 69535; fig. S1A) were designed. The single guide RNAs (sgRNAs) were synthesized by using the in vitro transcription EnGen Kit [New England Biolabs (NEB), E3322S]. DNA oligonucleotides for the sgRNA synthesis were generated with the NEB tool (http://nebiocalculator.neb.com/#!/sgrna) and ordered from Metabion. Following in vitro transcription, RNA was purified using the RNA Clean and Concentrator-25 from Zymo (R1017). The deletion allele injection mixes consisted of Cas9 protein (200 ng/μl) from IDT (Integrated DNA Technologies, 1081059) and sgRNA (50 ng/μl; two each) in a final volume of 5 μl of 1× OptiMEM.

The SgRNA/Cas9 mixture was electroporated into pronuclear stage of C57BL6/NCRL zygotes. For a single electroporation event, 50 one-cell stage embryos were filled in a glass chamber (1 mm) of a NEPA 21 electroporator (NEPA GENE Co. Ltd.), filled with 5 μl of OptiMEM (Thermo Fisher Scientific) containing sgRNA.RNP complexes following a standardized electroporation protocol. Injected zygotes were cultured overnight, and developed two-cell embryos were transferred into pseudopregnant [day 0.5 post-coital (pc)] CD-1 females on the day after the injection (on average, 16 embryos per recipient female). For genotype analysis, genomic DNA extracted from tissue samples was used for PCR with *Ten1*-specific primers (*Ten1* for aagctgggtatggtggctcctgcc and *Ten1* rev ttctctgtatagccttggtgtagc). The mutation was verified by Sanger sequencing.

### RNA QC

RNA was isolated from lung tissue using a Qiagen RNeasy Mini Kit (https://qiagen.com) according to the manufacturer’s instructions. cDNA was obtained using the ProtoScript II Reverse Transcriptase (https://international.neb.com). A 1:5 dilution of the cDNA was used for PCR with the primers *Ten1* Exon 2 CCAACGATTTGGATCCTGGTGCT and *Ten1* Exon 4 rev CTGGGGTAGGGAATCATATTCAT to detect the deletion of exon 3. The mutation was verified by Sanger sequencing.

### Housing and breeding

Mice were maintained in individually ventilated cages (IVC) with water and standard mouse chow according to the directive 2010/63/EU, German laws, and German Mouse Clinic (GMC) housing conditions (www.mouseclinic.de). All tests were approved by the responsible authority of the district government of Upper Bavaria, Bavaria (ROB-55.2-2532.Vet_02-18-117; ROB-55.2-2532.Vet_02-20-35). Animals were monitored daily by visual inspection of cages with maximum effort to minimize stress for the animals. If applicable, *Ten1* hom mice were housed with littermates after weaning, or alternatively, they were allowed to stay with their mothers until sacrifice. Body weights were taken on the day of sacrifice. Cohort breeding: The het matings were used for generation of hom mutants and WT littermate controls for tissue collection.

For P0.5 tissue and genotype distribution, 15 het x het matings were set up, yielding 107 animals in 13 litters (male *Ten1* WT, het, hom; female *Ten1* WT, het, hom: 7, 33, 13; 13, 27, 14). For survival analysis, 22 het x het matings were set up, yielding 111 animals in 17 litters (male *Ten1* WT, het, hom; female *Ten1* WT, het, hom: 10, 26, 22; 23, 28, 2). Mice were monitored daily, and an endpoint of death or welfare issue severe enough that killing was necessary was determined. Survival data included all hom mice and those WT littermates, which were not sacrificed as a tissue control (*Ten1* WT, hom: 33, 25). Additional mice were produced to collect tissue from P5, P9, and P19 to 22.

### μCT

For the skeleton analysis, datasets were acquired at 36-μm^3^ voxel resolution using a SkyScan 1176 micro-CT system (Bruker microCT, Kontich, Belgium). All scans were acquired with the following parameters: 50-kV source voltage, 500-μA source current, 60-ms exposure time, 360° rotation, 0.5° rotation step, 4 frame averages, and 0.5-mm aluminum filter. Image reconstruction was performed using NRecon software (Bruker microCT, Kontich, Belgium; version 1.7.5). Three-dimensional volume rendering was done with CTvox (Bruker microCT, Kontich, Belgium; version 3.3.1).

### Pathological analyses and IHC

Macroscopic paw images were captured with a DMS300 Digital Microscope System (Leica Microsystems). Following a complete dissection, histopathological analyses of formalin-fixed, paraffin-embedded, and H&E-stained 3-μm-thick sections were performed. For bone marrow histology, preincubation with Osteosoft mild decalcifier (Merck) was carried out (3 days for P5 and 7 days for P23 mice) after tissue fixation. Melanin was detected using a Fontana-Masson staining kit according to the manufacturer’s instructions (ab150669, Abcam).

A Leica BOND RX (Leica Biosystems) automated immunostainer was used for IHC. Heat-induced antigen retrieval was performed with citrate buffer (pH 6) for 30 min (AR9961, Leica Biosystems). Primary antibodies against Ki67 (1:500; ab15580, Abcam), p53 (1:400; CM5, Leica), p21 (1:200; ab107099, Abcam), pH2AX (1:2000; #2577, Cell Signaling Technology), pATM (S1981) (1:250; sc-47739, Santa Cruz Biotechnology), Sox9 (1:1000; ab185966, Abcam), CK15 (1:100; ab52816, Abcam), and calbindin (1:5000; ab229915, Abcam) were used, and the staining was detected with 3,3’-Diaminobenzidine (DAB) chromogen (Bond Polymer Refine Detection DAB). To detect apoptosis, TUNEL staining was performed using a kit (ab206386, Abcam) following the manufacturer’s instructions. For eye histology, the enucleated eyes were fixed in Davidson solution for 3 days and subsequently immersed in 80% ethanol until paraffin embedding. Tissue blocks were sectioned at 2-μm mid-sagittal sections on a HM355S microtome (Thermo Fisher Scientific, Germany) on superfrost slides and then stained by H&E. Microscopical slides were scanned using a Hamamatsu NanoZoomer 2.0HT digital scanner and visualized using NDP.view2 software (Hamamatsu Photonics). Double immunofluorescence was performed on cerebellum sections after EDTA (pH 9) antigen retrieval for 20 min (AR9640, Leica Biosystems) using calbindin (1:2000; ab229915, Abcam) and NeuN (1:100; ab104224, Abcam) primary antibodies. Secondary antibodies used were anti-rabbit Alexa488 (A11034, Life Technologies) and anti-mouse DyLight 550 (ab96880, Abcam). Images were captured using a BZ-X810 (Keyence) fluorescence microscope.

### Collection and processing of tissues for RT-qPCR, Western blot, and mass spectrometry–based analyses

The brain, liver, lung, and skin tissues were quickly isolated after animal sacrifice, snap frozen in liquid nitrogen, and stored at −80°C. Afterward, tissues were grinded to powder using a porcelain mortar and pestle maintained on dry ice, aliquoted, and stored at −80°C until further use.

### RNA isolation and RT-qPCR

Total RNA was extracted from mouse tissue powder by adding 1 ml of TRI-reagent (Merck) and following the manufacturer’s recommendations. RNA concentration was determined by a NanoDrop 2000c device (Thermo Fisher Scientific). The High-Capacity cDNA Reverse Transcription Kit (Thermo Fisher Scientific) was used for first-strand cDNA synthesis. RT-qPCR was performed on a QuantStudio 6 real-time PCR system (Thermo Fisher Scientific) using the AMPLIFYME SYBR Universal Mix (Blirt). For data analyses, the cycle threshold of the respective target gene was normalized to the cycle threshold of *Actb* (encoding β-actin). Primer sequences used are listed in table S3.

### Telomere length measurements

For the qPCR method, total genomic DNA was extracted from mouse tissue powder by adding 200 μl of DirectPCR Lysis Reagent (Viagen Biotech Inc.) and 0.5 μl of proteinase K solution (20 mg/ml). The suspension was incubated for 2 hours at 56°C with continuous shaking at 700 rpm. Afterward, the solution was incubated for 45 min at 85°C without shaking. Insoluble material was pelleted at 5000*g* for 2 min at 4°C, and the supernatant containing genomic DNA was used for telomere length measurement via SYBR Green based RT-qPCR on a QuantStudio 6 real-time PCR system with primers adopted from a protocol previously described (*98*). For data analyses, the cycle threshold of the telomeres was normalized to the cycle threshold of the single-copy gene 36B4.

For Q-FISH, paraffin-embedded tissue sections were deparaffinized and fixed with 4% formaldehyde, followed by digestion with pepsin/HCl and a second fixation with 4% formaldehyde. Slides were dehydrated with increasing concentrations of ethanol (70, 90, and 100%) and incubated with the telomeric (TTAGGG) probe labeled with Cy3 at 85°C for 3 min followed by 1 hour at room temperature in a wet chamber. The slides were extensively washed with 50% formamide and 0.08% TBS–Tween 20. Confocal microscopy was performed at room temperature with a laser scanning microscope (Leica TSC SP8) using a Plan Apo 63 Å–1.40 numerical aperture oil immersion objective (Leica HCX). Maximal projection of z-stack images generated using advanced fluorescence software Leica Application Suite (LAS) was analyzed with LAS X. The 4′,6-diamidino-2-phenylindole images were used to detect telomeric signals inside each nucleus.

### Western blot

Twenty micrograms of protein mixture derived from the brain, liver, or lung was separated in self-cast tris-glycin gels and transferred onto a 0.1-μm nitrocellulose membrane (GE Healthcare) afterward. Phosphate-buffered saline (PBS) containing 10% (w/v) skim milk (Carl Roth) was applied for 1 hour at room temperature to minimize unspecific binding. The respective primary antibody was diluted in PBS + 1% milk (w/v) solution and added to the membrane for overnight incubation at 4°C. After a series of washing steps using PBS and PBS + 0.1% Tween 20 (Carl Roth), the secondary antibody solution prepared in PBS + 0.5% milk (w/v) was applied for 90 min at room temperature. The membrane was washed again, and the detection of the immunoreactive target was completed by using the WesternBright enhanced chemiluminesence horseradish peroxidase (HRP) substrate (Advansta). ImageJ software (version 1.53q) was used for densitometric analysis, while the density of the target band was normalized to actin detected in the same lane. Primary antibodies used included pH2AX (S139) (1:1000; #2577, Cell Signaling Technology), pATR (S428) (1:000; #2853, Cell Signaling Technology), pATM (S1981) (1:1000; #4526, clone 1H11.E12, Cell Signaling Technology), and actin (1:10,000; #SKU0869100-CF, clone c4, MP Biomedicals). The goat anti-rabbit HRP-conjugated antibody (1:3000; W4011, Promega) and the goat anti-mouse HRP-conjugated antibody (1:10,000; P0447, Agilent Technologies) served as secondary antibodies.

### Sure-Quant mass spectrometry

Frozen mouse tissue powder was lysed in 200 μl of lysis buffer [50 mM Hepes (pH 7.4), 150 mM NaCl, 1 mM EDTA, 1.5% SDS, and 1 mM dithiothreitol; supplemented with 1× protease and phosphatase inhibitor cocktail (Thermo Fisher Scientific)]. Lysis was aided by repeated cycles of sonication in a water bath [6 cycles of 1 min sonication (35 kHz) intermitted by 2-min incubation on ice]. Protein lysates were reduced, alkylated, and then processed by a modified filter-aided sample preparation protocol as previously described (*99*). Samples were digested overnight with trypsin (1:20) at 30°C and precipitated using an equal volume of 2 M KCl for depletion of residual detergents. Tryptic peptides were cleaned and desalted on C18 stage tips and then resuspended in 20 μl of 1% formic acid for SureQuant-based mass spectrometry (MS) analysis as previously described (*100*).

SureQuant analysis was performed as previously described (*101*). Peak area ratios of endogenous light peptides and corresponding heavy internal standard (IS) peptides for the six selected product ions were exported from Skyline software v21.1.0.278 (*102*). Quantitation was based on three selected product ions to balance specificity with the ability to retain lowly abundant targets.

### Randomization, blinding, and data exclusion

For molecular analyses, experimenters were blinded to the genotype. Randomization and blinding were done for general analyses of het and control animals, but hom mutants were obviously identifiable macroscopically. Data exclusion, if done, was only performed for quality control reasons.

### Statistics

The data are presented as means ± SEM if not denoted elsewise. Two-sided unpaired *t* tests were applied to compare differences between *Ten1* hom versus control mice at a given age stage. If animals of different age groups were included, the data were analyzed by two-way analyses of variance (ANOVAs) with the between-subjects factors age and genotype, followed by Tukey post hoc tests. Statistical analyses were performed using GraphPad (v9.3.0), and the significance threshold was set at *P* < 0.05.
