# Supplementary material for: Loss of Ten1 in mice induces telomere shortening and models human dyskeratosis congenita
Source: Sci Adv. 2025 Apr 11;11(15):eadp8093. doi: 10.1126/sciadv.adp8093 (PMC11988282; doi:10.1126/sciadv.adp8093)
Supplement: Supplementary file 1 — Figs. S1 to S13 Tables S1 to S4 [file sciadv.adp8093_sm.pdf]

Science Advances
AAAS

Supplementary Materials for

Loss of Ten1 in mice induces telomere shortening and models human dyskeratosis congenita

Adrián Sanz-Moreno *et al.*

Corresponding author: Martin Hrabě de Angelis, martin.hrabedeangelis@helmholtz-munich.de

Sci. Adv. **11**, eadp8093 (2025)
DOI: 10.1126/sciadv.adp8093

This PDF file includes:

Figs. S1 to S13
Tables S1 to S4

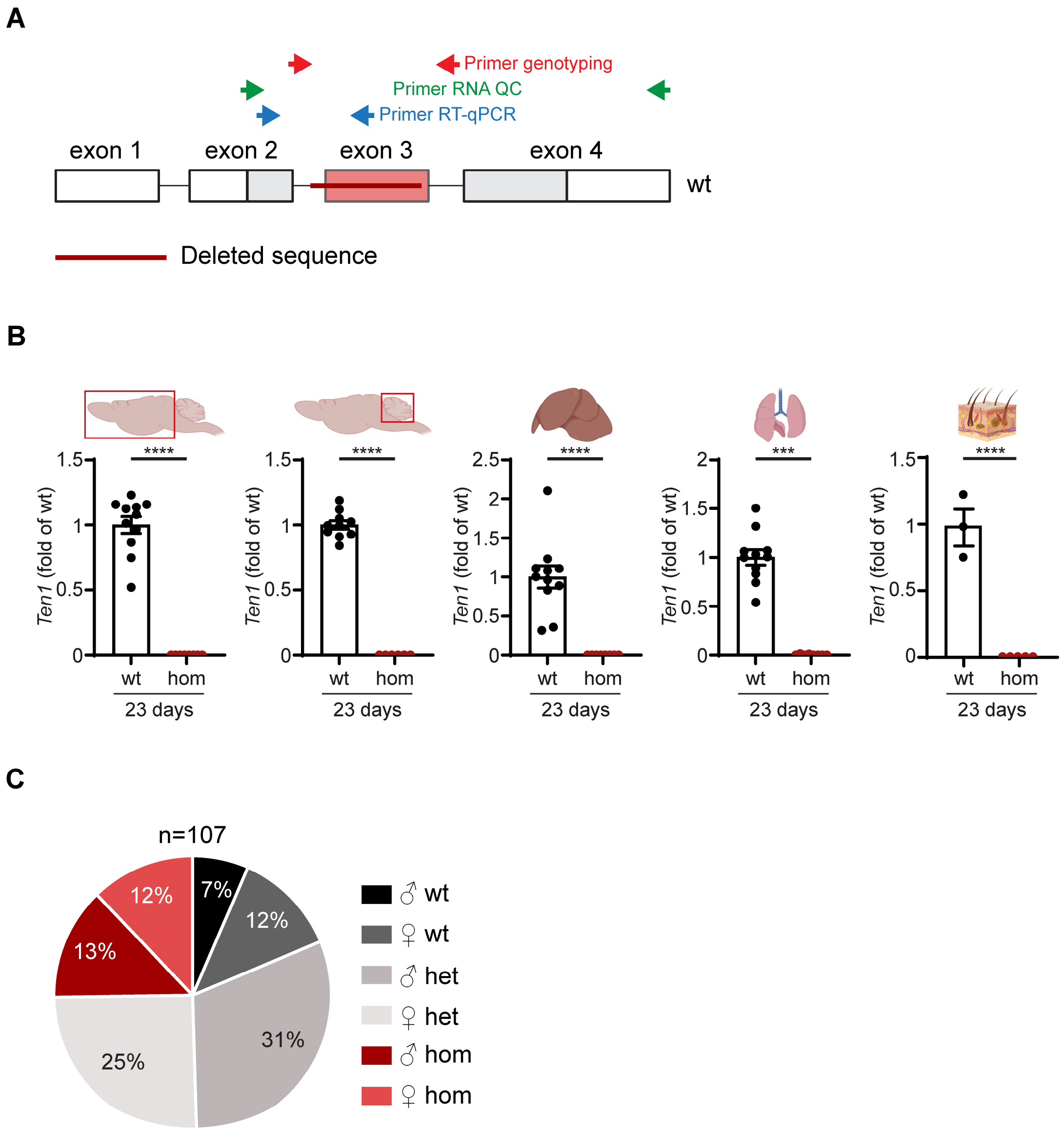

Fig. S1: *Ten1* mouse model was established with knockout of major part of exon 3. (A) Depiction of the deletion in *Ten1* exon 3 specifying the different primers used. (B) RT-qPCR of wildtype *Ten1* mRNA transcripts in cerebrum, cerebellum, liver, lung, and skin. (C) Genotype distribution at P0.5 in a cohort of 107 animals. Figure elements in (B) were created with BioRender.

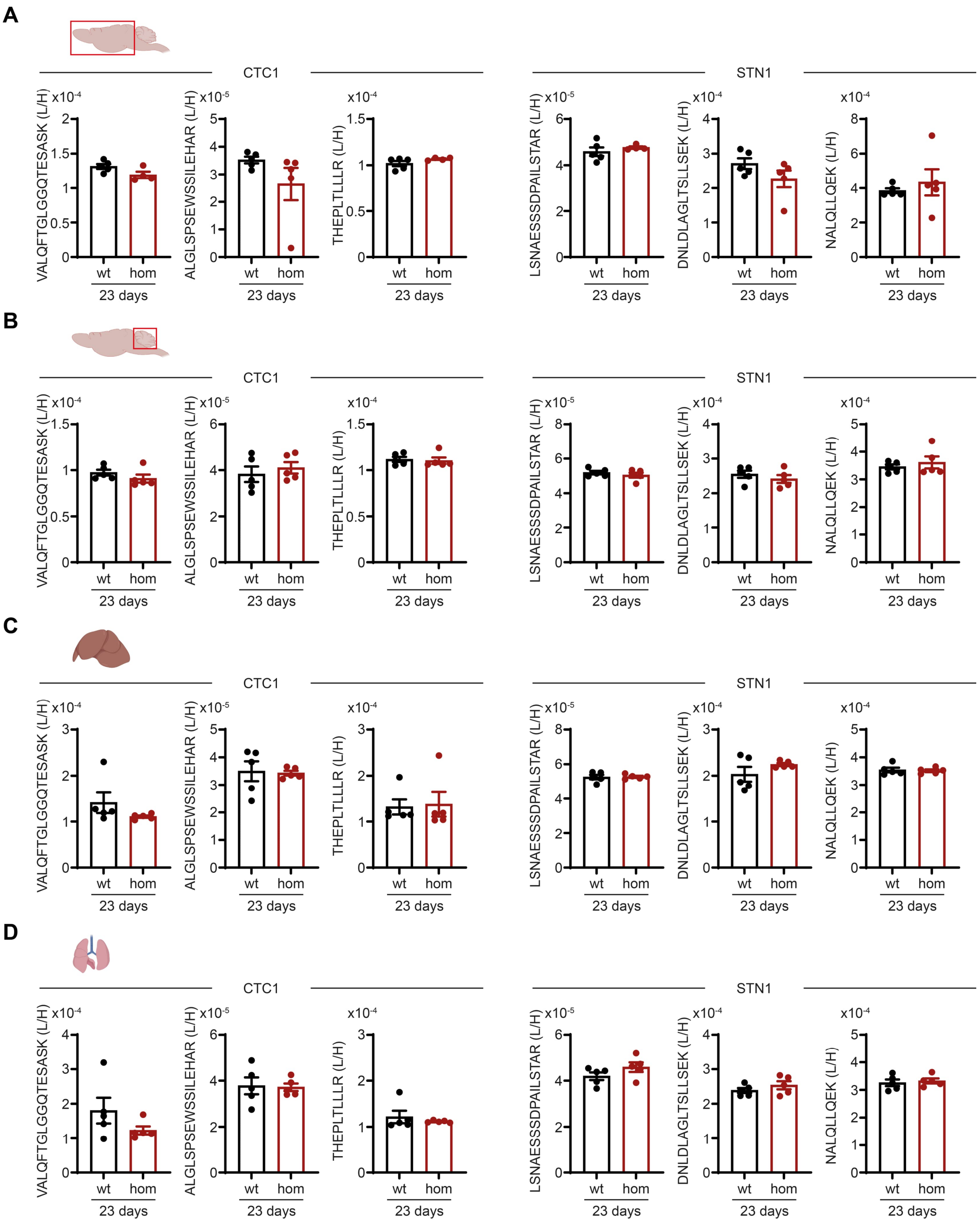

Fig. S2: Protein abundance of the TEN1 interaction partners, CTC1 and STN1, measured by Sure-Quant mass spectrometry in (A) cerebrum, (B) cerebellum, (C) liver, and (D) lung. Figure elements were created with BioRender.

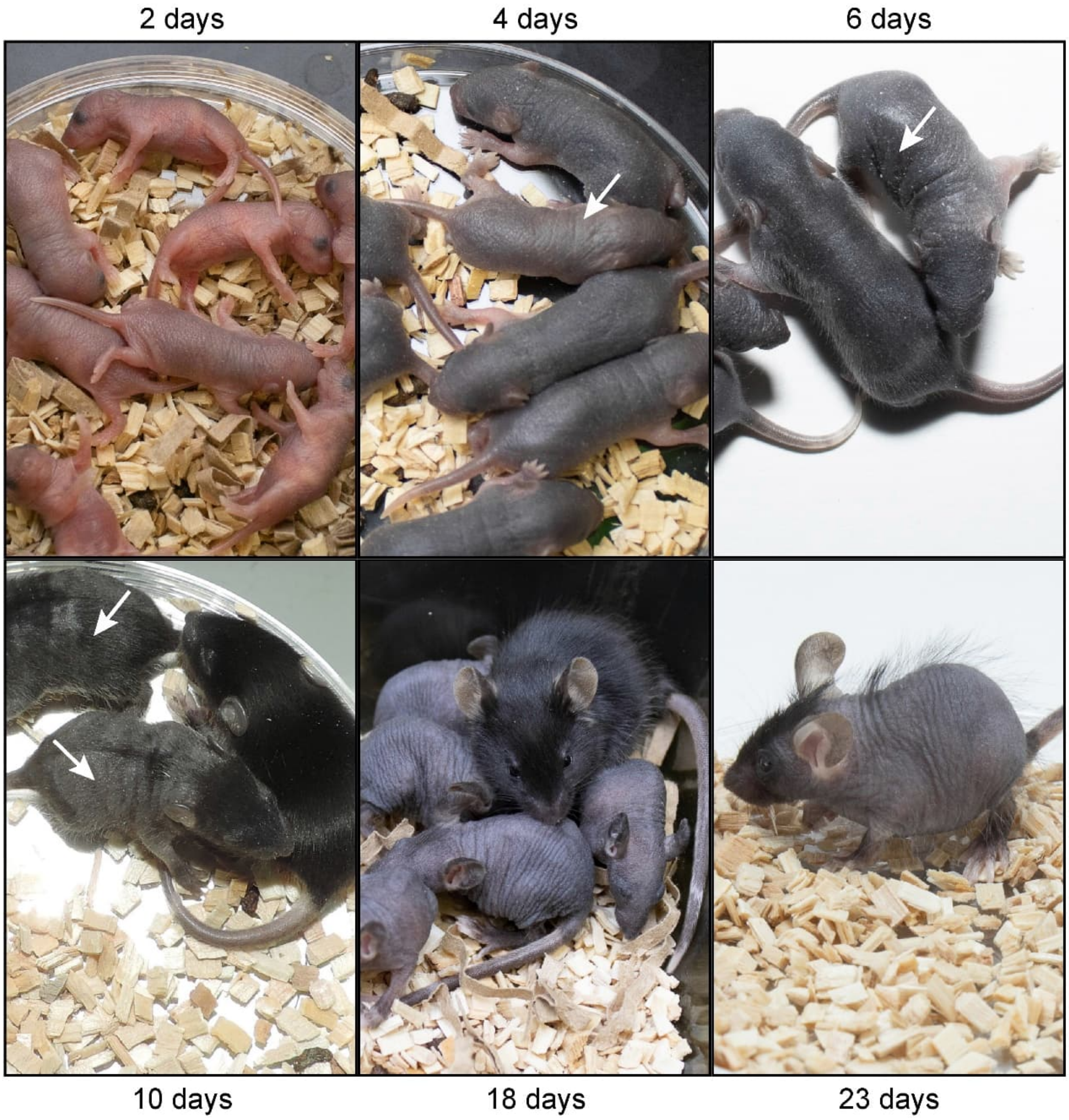

Fig. S3: Representative pictures of *Ten1* hom mice (arrows) and littermates at different ages.

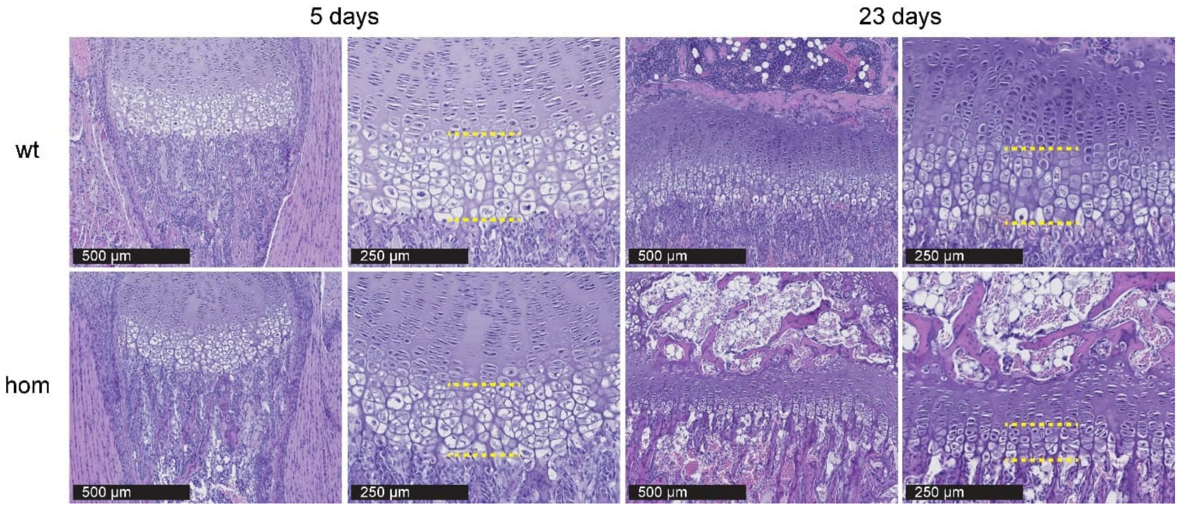

Fig. S4: Decreased number of hypertrophic chondrocytes in the tibial growth plates of *Ten1* hom mice. Representative pictures of H&E-stained tibial growth plate sections at P5 (*n* wt/hom: 2/3) and P23 (*n* wt/hom: 3/3).

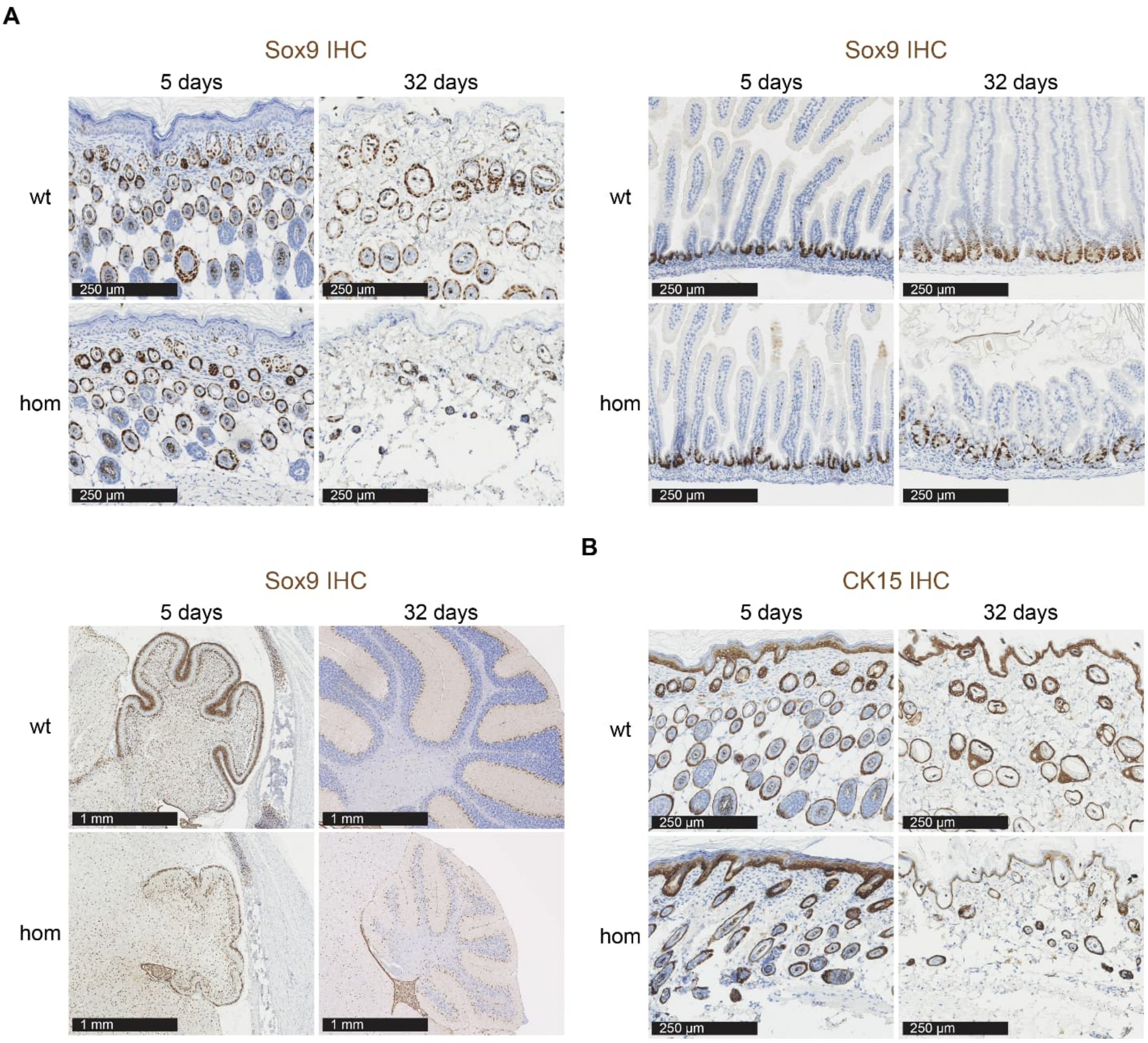

Fig. S5: Analysis stem cell marker expression in skin, small intestine and cerebellum of *Ten1* hom animals. (A) Sox9 IHC in skin, small intestine, and cerebellum at P5 and P32. (B) CK15 IHC in skin at P5 and P32. For (A) and (B): *n* wt/hom: P5 2/4; P32 1/1.

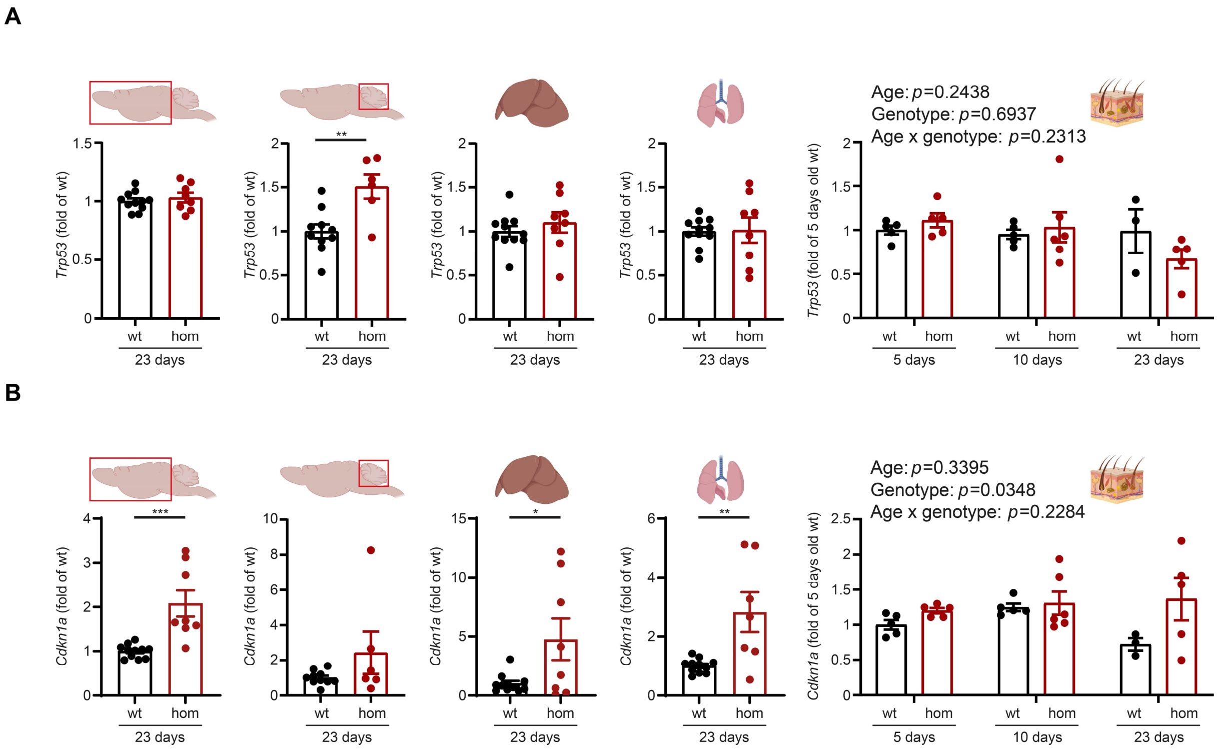

Fig. S6: *Trp53* and *Cdkn1a* expression levels by qPCR. *Trp53* (A) and *Cdkn1a/p21Cip* (B) gene expression levels upon *Ten1* deletion in cerebrum, cerebellum, liver, lung, and skin at the indicated time points. Figure elements were created with BioRender.

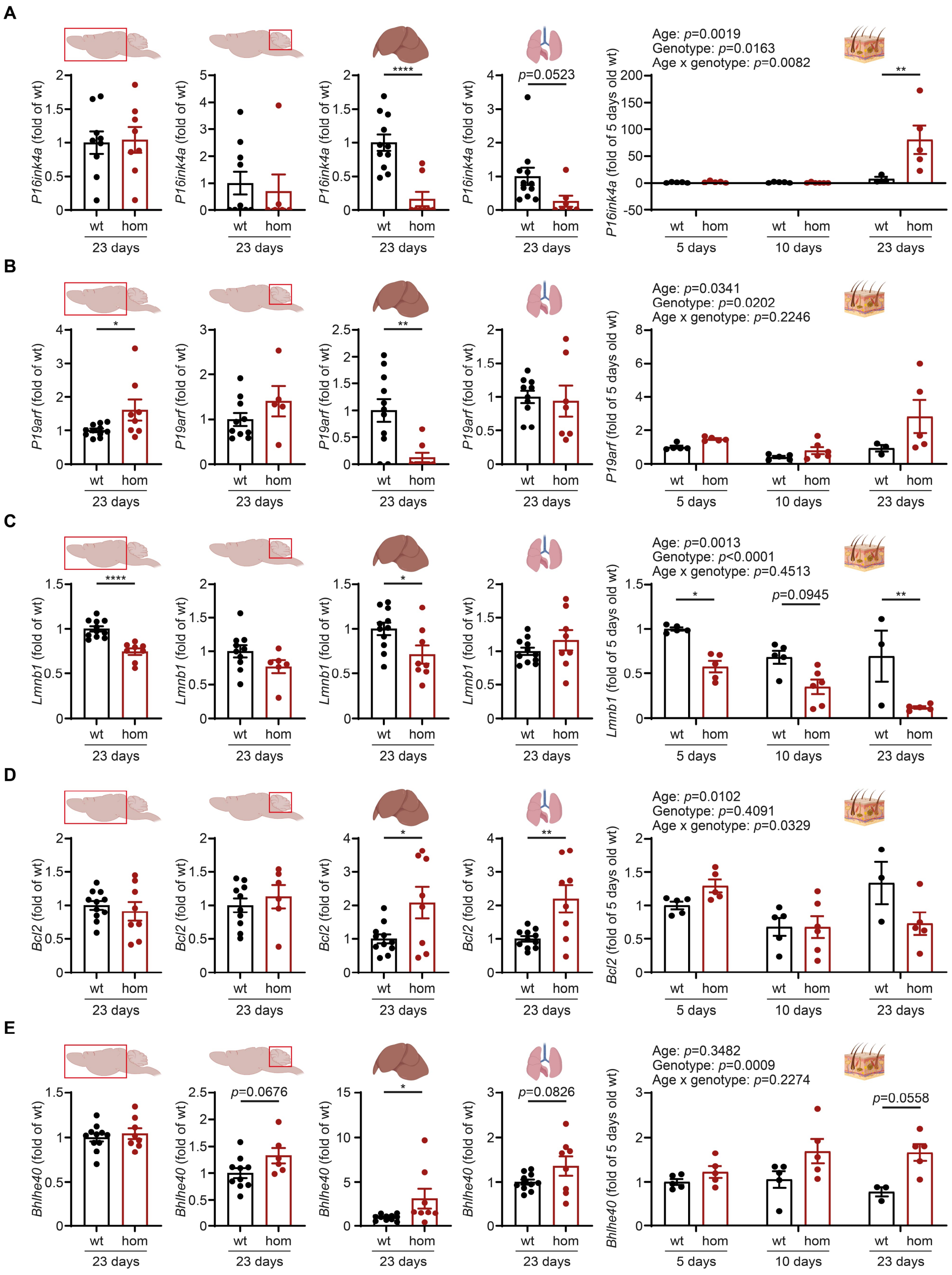

Fig. S7: Gene expression analysis of cellular senescence markers by qPCR. Transcripts of (A) *p16Ink4a*, (B) *p19Arf*, (C) *Lmnb1*, (D) *Bcl2*, and (E) *Bhlhe40* were measured by RT-qPCR in cerebrum, cerebellum, liver, lung, and skin isolated from *Ten1* hom vs. control mice. Figure elements were created with BioRender.

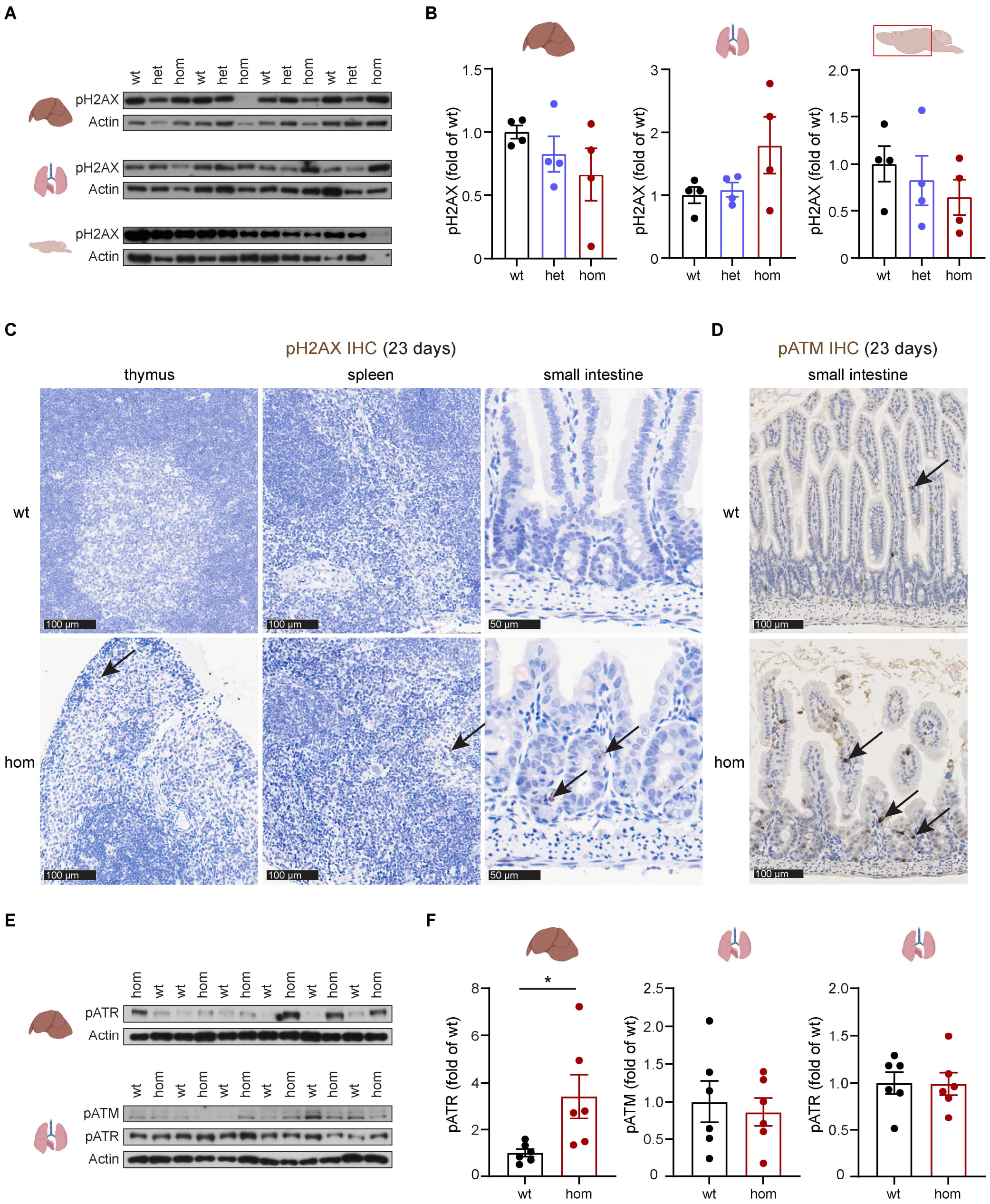

Fig. S8: DNA damage analyses at P23. (A) Representative western blot images and (B) quantitation of pH2AX are shown in liver, lung, and cerebrum (*n* wt/het/hom: 4/4/4). (C) IHC of pH2AX in different tissues (*n* wt/hom: 6/7). Arrows indicate example cells showing positive immunoreactivity. (D) IHC of pATM in small intestine (*n* wt/hom: 6/6). (E) Representative western blot images and (F) quantitation of pATR and pATM in liver and lung (*n* wt/hom: 6/6). Figure elements were created with BioRender.

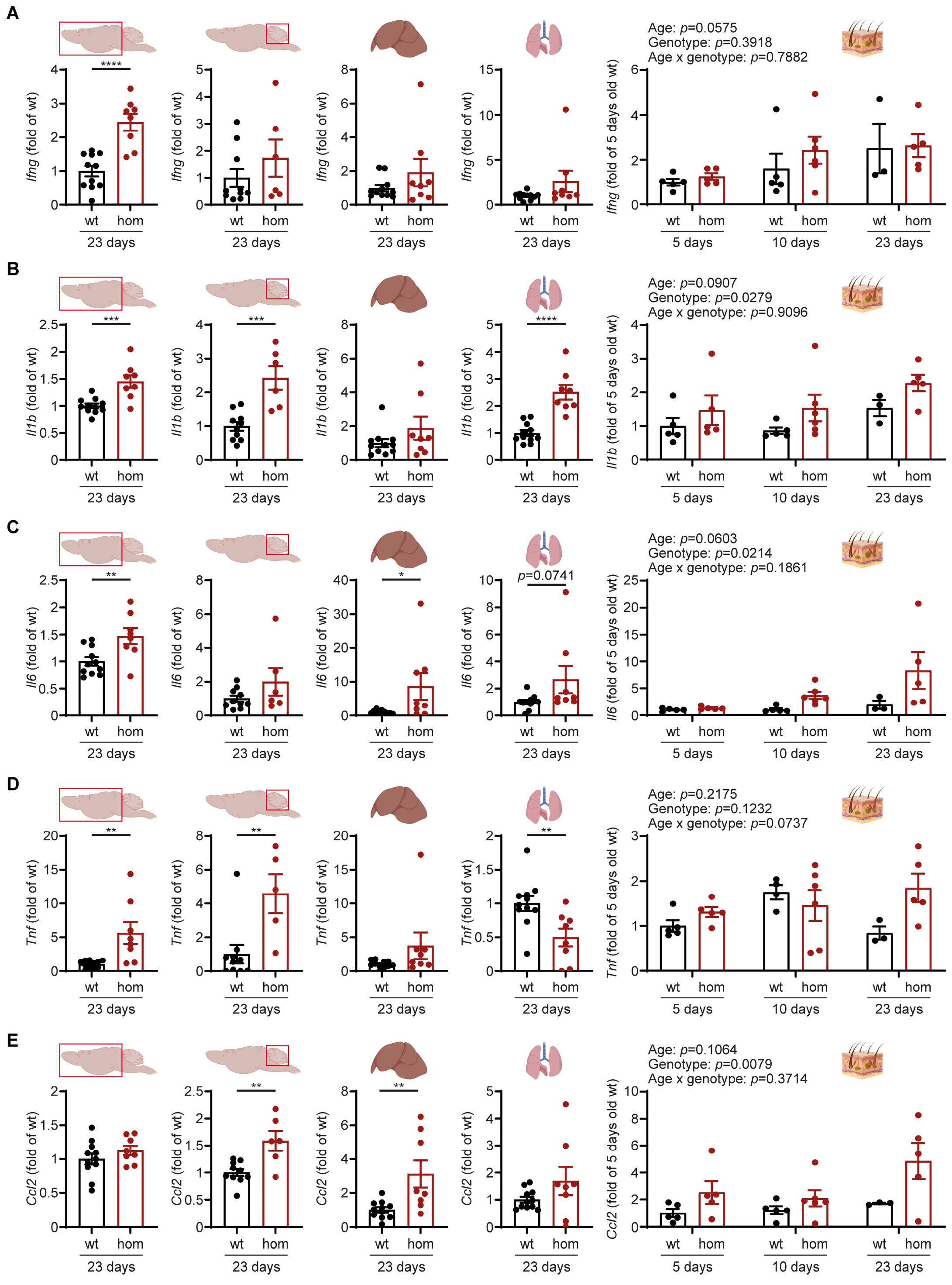

Fig. S9: Effects of *Ten1* loss on gene expression levels of pro-inflammatory cytokines. Gene expression levels of (A) *Ifng*, (B) *Il1b*, (C) *Il6*, (D) *Tnf*, and (E) *Ccl2* were determined in cerebrum, cerebellum, liver, lung, and skin derived from *Ten1* hom vs. control mice. Figure elements were created with BioRender.

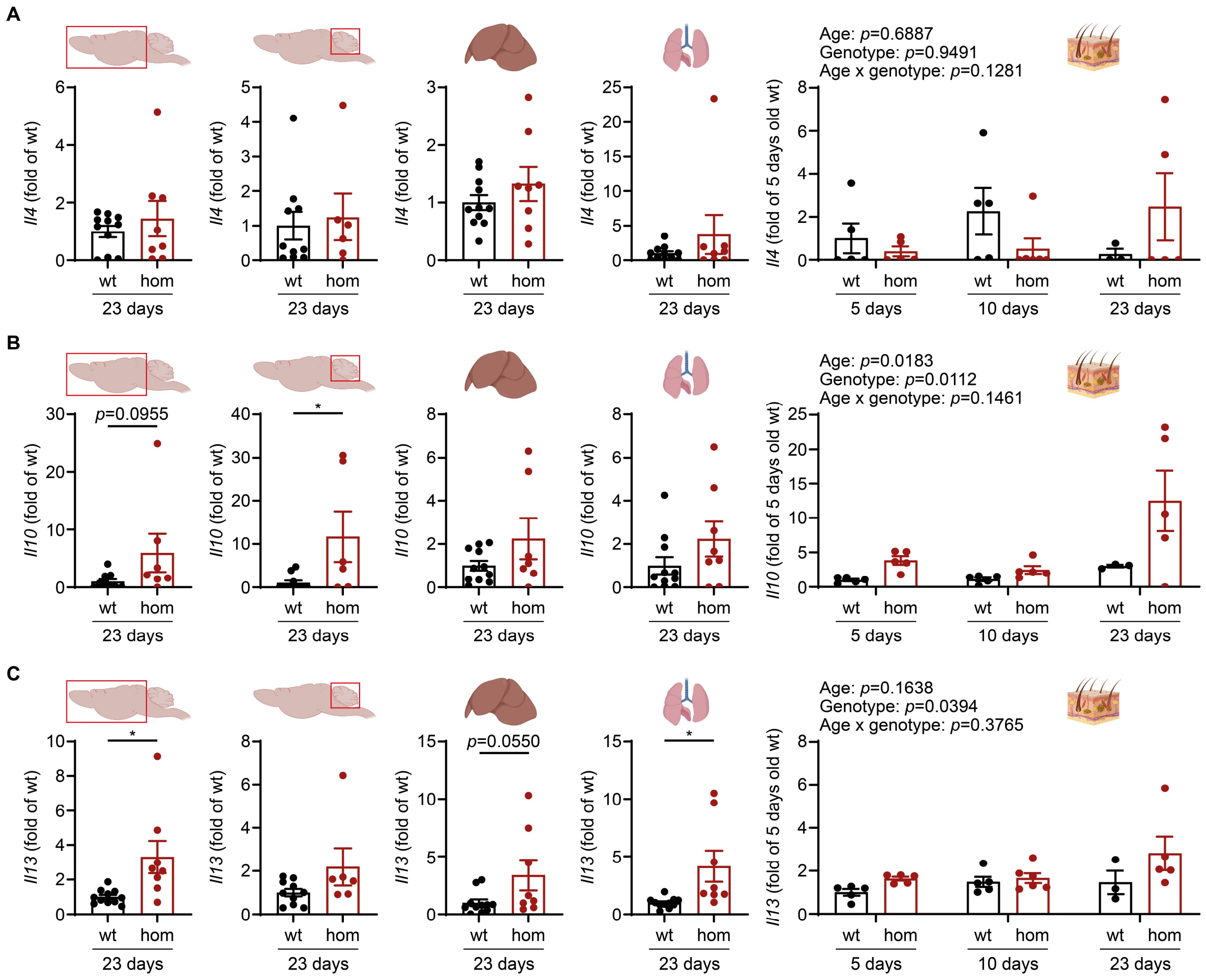

Fig. S10: Effects of Ten1 loss on gene expression levels of anti-inflammatory cytokines. The mRNA abundance of three anti-inflammatory cytokines (A) Il4, (B) Il10, and (C) Il13 was compared between Ten1 hom vs. control animals in cerebrum, cerebellum, liver, lung, and skin. Figure elements were created with BioRender.

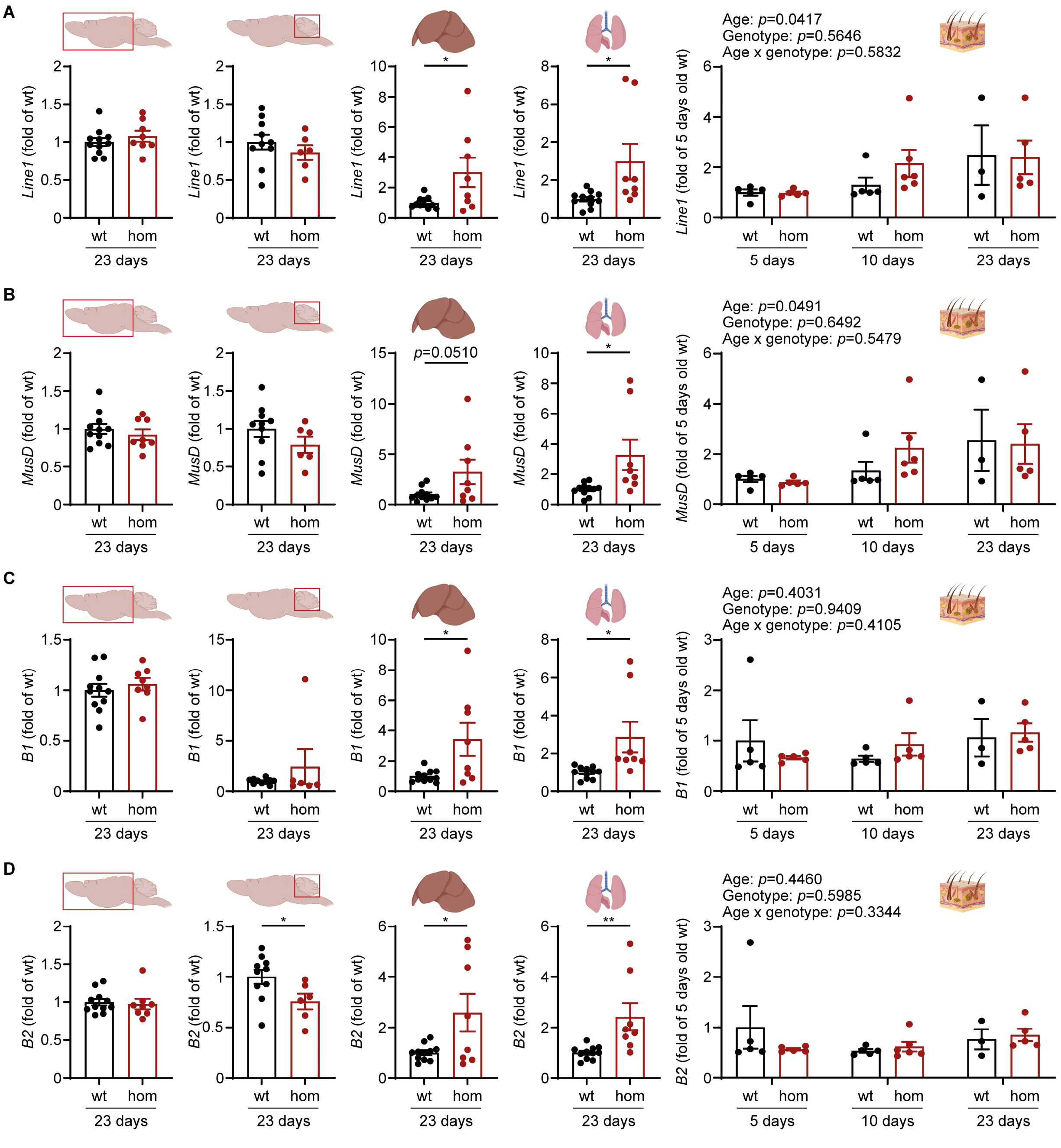

Fig. S11: Transcription of transposable elements by qPCR. Transcriptional activities of (A) Line1, (B) MusD, (C) B1, and (D) B2 transposon classes were determined by RT-qPCR in cerebrum, cerebellum, liver, lung, and skin isolated from Ten1 hom vs. control mice. Figure elements were created with BioRender.

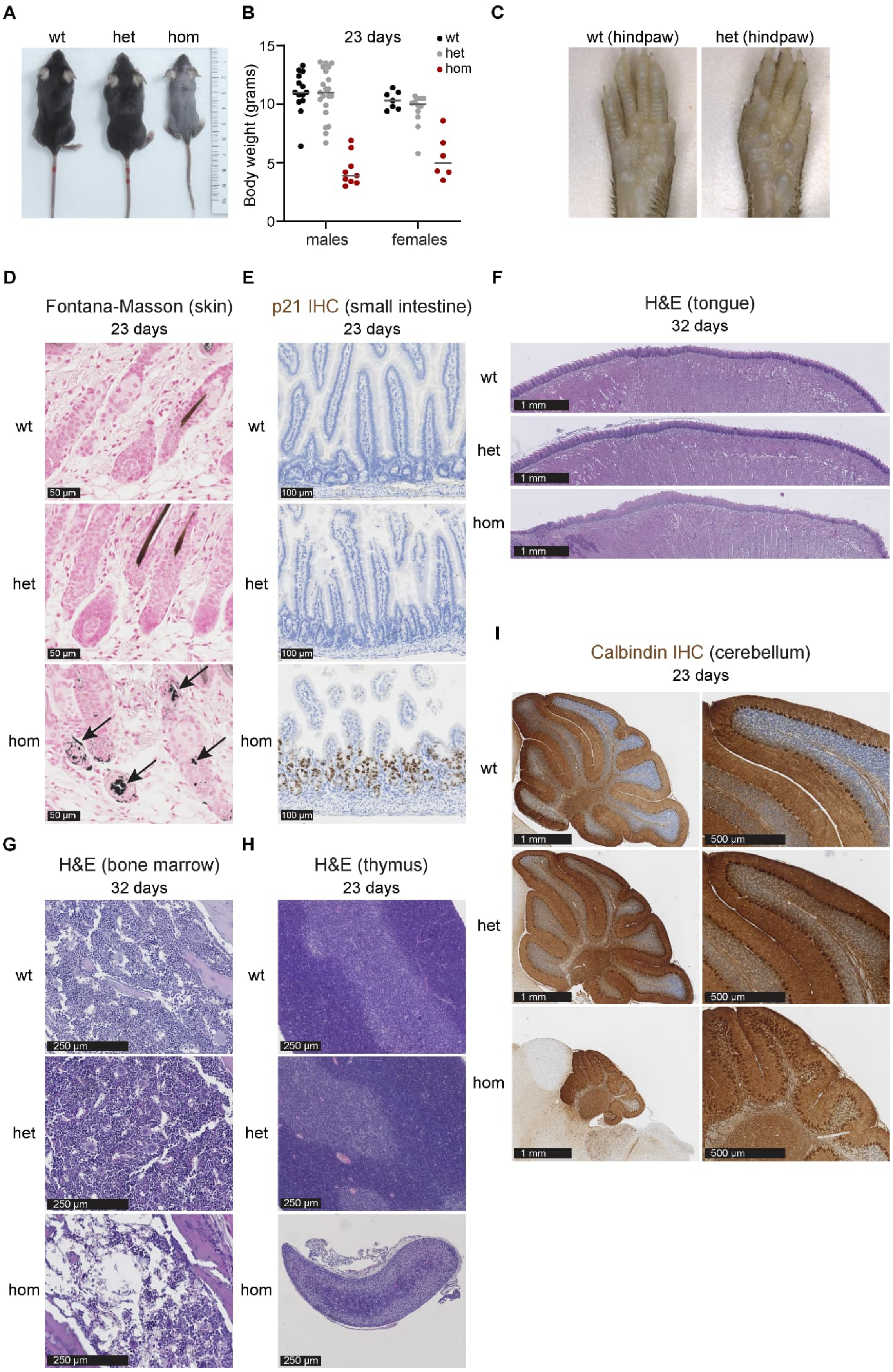
A
wt
het
hom
B
23 days
wt
het
hom
Body weight (grams)
15
10
5
0
males
females
C
wt (hindpaw)
het (hindpaw)
D
Fontana-Masson (skin)
23 days
wt
het
hom
50 µm
E
p21 IHC (small intestine)
23 days
wt
het
hom
100 µm
F
H&E (tongue)
32 days
wt
het
hom
1 mm
G
H&E (bone marrow)
32 days
wt
het
hom
250 µm
H
H&E (thymus)
23 days
wt
het
hom
250 µm
I
Calbindin IHC (cerebellum)
23 days
wt
het
hom
1 mm
500 µm

Fig. S12: *Ten1* het mice do not show the described phenotypes up to 1 month of age. (A) Representative picture of age-matched (P23) wt, het, and hom mice. (B) Body weight at P23. (C) Macroscopical pictures of hindpaws (*n* wt/het: 5/4) and (D) Fontana-Masson staining (*n* wt/het/hom: 6/6/6) revealed no skin hyperpigmentation in hets at P23. (E) p21 IHC in small intestine (*n* wt/het/hom: 6/6/7). Neither (F) tongue hyperkeratosis nor (G) aplastic anemia were found in het animals up to 32 days of age as shown by H&E staining (only n=1 at this late time point). (H) Thymus H&E staining at P23 show no abnormalities in hets (*n* wt/het/hom: 6/6/7). (I) Cerebellar hypoplasia was not found in P23 het animals as shown in these Calbindin IHC representative cerebellum images taken at the same magnification (*n* wt/het/hom: 6/6/7).

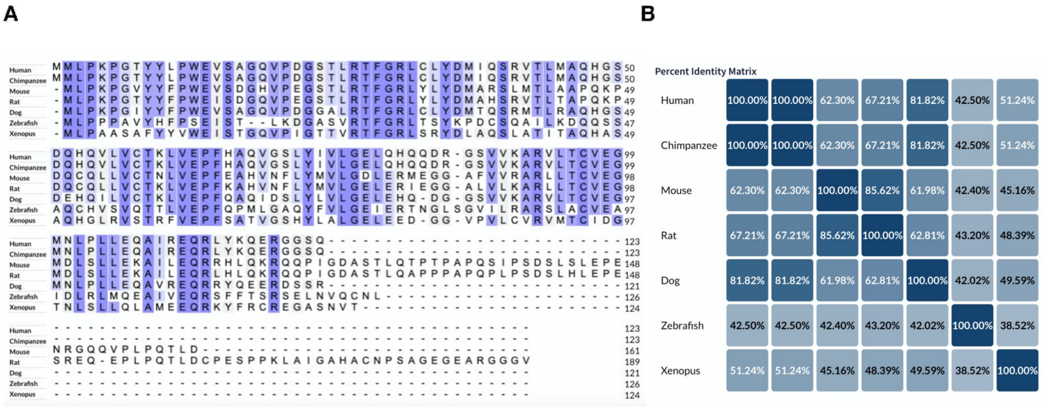

Fig. S13: TEN1 homology between different species (A) by sequence or (B) represented in a percent identity matrix.

Table S1. Effects of *Ten1* ablation on gene expression levels of cell cycle regulators in cerebrum, cerebellum, liver, and lung.

Ten1 homozygous mutant vs. wildtype control animals at 23 days of age were compared using two-sided t-tests.

Target gene	Cerebrum	Cerebellum	Liver	Lung
Ccna1	→	↑	→	↓
Ccna2	→	↑	↓	↓
Ccnb1	→	→	→	→
Ccnb2	→	↓	↓	↓
Ccnc	→	→	→	→
Ccnd1	→	→	↓	↓
Ccnd2	→	↑	→	↓
Ccnd3	→	→	→	→
Ccne1	→	↑	→	→
Ccne2	→	↓	→	→

→ not significant, ↑ increased, ↓ decreased in *Ten1* hom mice

Table S2. Effects of *Ten1* ablation on gene expression levels of cell cycle regulators in the skin.

Ten1 homozygous mutant vs. wildtype control animals at 5 days, 10 days, and 23 days of age were compared using two-way ANOVA.

Target gene	Age x genotype interaction	Genotype	Age
Ccna1	No	→	→
Ccna2	No	↓	→
Ccnb1	No	→	→
Ccnb2	No	→	↓
Ccnc	No	→	→
Ccnd1	No	→	↓
Ccnd2	No	→	↓
Ccnd3	No	↑	↓
Ccne1	No	↓	↓
Ccne2	Yes	↓	↓

→ not significant, ↑ increased, ↓ decreased in *Ten1* hom mice

Table S3. Primer sequences used for real-time quantitative PCR analyses.

Category	Target gene	Primer forward	Primer reverse
**Cellular proliferation**	*Ccna1*	GGGTGTTGACTGAAAATGAGC	CACGTTTGGCTGGTTCATTG
	Ccna2	GTCCTTGCTTTTGACTTGGC	ACGGGTCAGCATCTATCAAAC
	Ccnb1	CTGACCCAAACCTCTGTAGTG	CCTGTATTAGCCAGTCAATGAGG
	Ccnb2	CCTCAGAACACCAAAGTACCAG	CCTTCATGGAGACATCCTCAG
	Ccnc	GCATTTGTATCAGGGCAAGC	GAAACTTTAGGTCCTTTTGGCG
	Ccnd1	GCCCTCCGTATCTTACTTCAAG	GCGGTCCAGGTAGTTCATG
	Ccnd2	GTGTTCCTATTTCAAGTGCGTG	AGCCAAGAAACGGTCCAG
	Ccnd3	GCGTGCAAAAGGAGATCAAG	GATCCAGGTAGTTCATAGCCAG
	Ccne1	GCGAGGATGAGAGCAGTTC	AAGTCCTGTGCCAAGTAGAAC
	Ccne2	GACGTTCATCCAGATAGCTCAG	TCCCATTCCAAACCTGAAGC
	Mki67	TGCCCGACCCTACAAAATG	GAGCCTGTATCACTCATCTGC
**Cellular senescence**	*Cdkn2a/p16Ink4a*	CCCAACGCCCCGAACT	GCAGAAGAGCTGCTACGTGAA
	Cdkn2a/p19Arf	CTCTGGCTTTCGTGAACATG	TCGAATCTGCACCGTAGTTG
	Cdkn1a/p21Cip	CAGATCCACAGCGATATCCAG	AGAGACAACGGCACACTTTG
	Lmnb1	CCTCAGAGATGAACACTTCCAC	TCCTTTCCAAACACGCTCTAG
	Bcl2	GAGGAACTCTTCAGGGATGG	GTTCAGGTACTCAGTCATCCAC
	Bhlhe40	CAACCACCTCCTACCTGC	GTGCGGCAGTTTGTAAGTTTC
**Inflammation**	*Ccl2*	AAGAGATCAGGGAGTTTGCT	CTGCCTCCATCAACCACTTT
	Ifng	CTTTGGACCCTCTGACTTGAG	TCAATGACTGTGCCGTGG
	Il1b	GAAGAAGAGCCCATCCTCTG	TCATCTCGGAGCCTGTAGTG
	Il4	GCATTTTGAACGAGGTCACAG	TGGAAGCCCTACAGACGAG
	Il6	AGTCCGGAGAGGAGACTTCA	ATTTCCACGATTTCCCAGAG
	Il10	AGCCGGGAAGACAATAACTG	GGAGTCGGTTAGCAGTATGTTG
	Il13	ACCAAAATCGAAGTAGCCCAC	GCAAAGTCTGATGTGAGAAAGG
	Tnf	CTTCTGTCTACTGAACTTCGGG	CAGGCTTGTCACTCGAATTTTG
**Transposons**	*Line1*	GCGGTTCCTCAGAAAATTGG	TGCCAGGAGAGGTATTGCT
	MusD	ATAGAGGCCGCTTCTTTGC	TGAGACTCCACCAAATGTCC
	B1	CATGGTGGCGCACGCCTTTAATCC	CCAGGCTGGCCTCGAACTCAGAAA
	B2	GGGCTGGAGAGATGGCTCAGTGGT	GCCACCATGTGGTTGCTGGGAATTG
**Ten1**	*Ten1*	CCTGGTGCTGACAATGTTGC	GGAGCGTGCCATGTCATAGA
**Reference**	*Actb*	CCCTGAAGTACCCCATTGAAC	CCATGTCGTCCCAGTTGGTAA

Table S4. Peptide sequences used for Sure-Quant mass spectrometry-based analyses.

Protein/Unique Peptide (a,c)	Precursor ion (m/z)	Product ions used for quantitation (b)	Retention times (min)
**CST complex subunit CTC1**			
VALQFTGLGGQTESASK (light)	565.296698+++	(y9, y6, y4)	34.79
**VALQFTGLGGQTESASK (heavy)**	**567.968097+++**	**(y9, y6, y4)**	**34.76**
ALGLSPSEWSSILEHAR (light)	618.323247+++	(y9, y8, y7)	42.52
**ALGLSPSEWSSILEHAR (heavy)**	**621.659336+++**	**(y9, y8, y7)**	**43.01**
THEPLTLLLR (light)	596.856055++	(y8, y7, b2)	33.12
**THEPLTLLLR (heavy)**	**601.860189++**	**(y8, y7, b2)**	**33.41**
**CST complex subunit STN1**			
LSNAESSSDPAILSTAR (light)	859.931405++	(y13, y12, y8)	27.56
**LSNAESSSDPAILSTAR (heavy)**	**864.935539++**	**(y13, y12, y8)**	**27.45**
DNLDLAGLTSLLSEK (light)	794.92506++	(y10, y9, b2)	44.36
**DNLDLAGLTSLLSEK (heavy)**	**798.932159++**	**(y10, y9, b2)**	**44.33**
NALQLLQEK (light)	528.806031++	(y7, y5, b2)	31.03
**NALQLLQEK (heavy)**	**532.81313++**	**(y7, y5, b2)**	**31.02**

[a]Heavy peptides are labelled at the C-terminal of Arginine (R) or Lysine (K) and highlighted in bold.
[b]Fragments used for quantification are indicated and they are singly charged.
[c]Quantitation is based on the L/H ratios of the peptides.
